# Supplementary material for: Factors associated with food consumption and dietary diversity among infants aged 6–18 months in Ashanti Region, Ghana
Source: PLoS One. 2023 Nov 30;18(11):e0294864. doi: 10.1371/journal.pone.0294864 (PMC10688859; doi:10.1371/journal.pone.0294864)
Supplement: S1 Table — (PDF) [file pone.0294864.s001.pdf]

S1A Table : Food group consumption by age groups and dietary diversity of child

Age group (Months)	Inadequate DDS (< 9 food groups)	Adequate DD (≥ 9 food groups)	X^2	P-value
**Bread, rice, noodles**				
6 – 11	324 (61.0)	302 (39.0)	212.94	<0.001*
12 – 18	240 (45.5)	287 (54.5)		
**Cassava, Plantain, cocoyam**				
6 – 11	75 (58.1)	54 (41.9)	119.11	<0.001*
12 – 18	75 (37.7)	128 (62.3)		
**Porridge**				
6 – 11	448 (69.0)	201 (31.0)	55.26	<0.001*
12 – 18	234 (47.5)	259 (52.5)		
**Any oils (margarine, butter, etc**				
6 – 11	137 (50.2)	136 (49.8)	275.46	<0.001*
12 – 18	95 (30.1)	221 (69.9)		
**Sugary foods (sweet, chocolate)**				
6 – 11	158 (54.5)	132 (45.5)	210.84	<0.001*
12 – 18	108 (33.3)	216 (66.7)		
**Infant formula**				
6 – 11	229 (63.1)	134 (36.9)	43.4	<0.001*
12 – 18	66 (36.1)	117 (63.9)		
**Tinned powdered or fresh animal milk**				
6 – 11	152 (54.1)	129 (45.9)	213.44	<0.001*
12 – 18	334 (52.5)	302 (47.5)		
**Eggs**				
6 – 11	194 (54.3)	163 (45.7)	270.5	<0.001*
12 – 18	170 (38.8)	268 (61.2)		
**Fresh or dried fish**				
6 – 11	237 (55.5)	190 (44.5)	253.91	<0.001*

12 – 18	209 (43.4)	273 (56.6)		

Frequency (percentage), Fisher's exact test P values reported, p-values are significant at p < 0.05

S1B Table: Food group consumption by age groups and dietary diversity of child

Age group (Months)	Inadequate DD (< 9 food groups)	Adequate DD (≥ 9 food groups)	X^2	P-value
**Organ meat**				
6 – 11	27 (32.5)	56 (67.5)	201.3	<0.001*
12 – 18	17 (13.6)	108 (86.4)		
**Any meat**				
6 – 11	51 (35.7)	92 (64.3)	302.42	<0.001*
12 – 18	58 (24.0)	184 (76.0)		
**Foods made from beans, lentils, etc.**				
6 – 11	76 (36.5)	132 (63.5)	140.82	<0.001*
12 – 18	44 (20.1)	175 (79.9)		
**Yogurt or cheese**				
6 – 11	84 (43.3)	110 (56.7)	309.94	<0.001*
12 – 18	48 (20.3)	189 (79.7)		
**Fruits**				
6 – 11	190 (53.1)	168 (46.9)	303.33	<0.001*
12 – 18	131 (34.0)	254 (66.0)		
**Carrots or sweet potato**				
6 – 11	16 (21.3)	59 (78.7)	173.71	<0.001*
12 – 18	15 (16.5)	76 (83.5)		
**Dark green leafy vegetables**				
6 – 11	206 (53.8)	177 (46.2)	268.36	<0.001*
12 – 18	131 (35.4)	239 (64.6)		
**Ripe mangoes or pawpaw**				
6 – 11	17 (20.7)	65 (79.3)	211.84	<0.001*

12 – 18	21 (17.8)	97 (82.2)		
**Any other fruits and vegetables**				
6 – 11	116 (44.1)	147 (55.9)	81.95	<0.001*
12 – 18	80 (30.9)	179 (69.1)		

Frequency (percentage), Fisher's exact test P values reported, p-values are significant at p < 0.05
