# Supplementary material for: Factors associated with food consumption and dietary diversity among infants aged 6–18 months in Ashanti Region, Ghana
Source: PLoS One. 2023 Nov 30;18(11):e0294864. doi: 10.1371/journal.pone.0294864 (PMC10688859; doi:10.1371/journal.pone.0294864)
Supplement: S2 Table — (PDF) [file pone.0294864.s002.pdf]

S2A Table: Binary logistic regression analysis showing the predictors of infant dietary diversity.

Variables in the Equation

		B	S.E.	Wald	df	Sig.	Exp(B)	95% C.I.for EXP(B)	
								Lower	Upper
Step 1[a]	educational_level	.293	.177	2.750	1	.097	1.340	.948	1.895
	INCOMESTATUS	-.215	.225	.915	1	.339	.807	.519	1.253
	Childs_age(1)	.928	.288	10.375	1	.001	2.529	1.438	4.448
	resident_REGRESSION			1.311	2	.519			
	resident_REGRESSION (1)	.628	1.399	.201	1	.654	1.873	.121	29.070
	resident_REGRESSION (2)	1.652	1.471	1.261	1	.261	5.217	.292	93.254
	employment_REGRESSI ON(1)	.105	1.155	.008	1	.928	1.110	.115	10.676
	WHEN_GIVE_FAMILY_FO OD_GRADE(1)	.634	.277	5.220	1	.022	1.885	1.094	3.246
	HOW_MUCH_COMPLEM ENTARY_FOOD_SHOUL D_A_CHILD_EAT_AT_6M ONTHS(1)	-.049	.268	.033	1	.855	.952	.563	1.610
	HOWMANYTIMESFEEDA CHILDFROM9TO12MONT HS(1)	-.038	.439	.007	1	.931	.963	.407	2.278
	Constant	-.709	.634	1.248	1	.264	.492		

a. Variable(s) entered on step 1: Childs_age, resident_REGRESSION, employment_REGRESSION, WHEN_GIVE_FAMILY_FOOD_GRADE, HOW_MUCH_COMPLEMENTARY_FOOD_SHOULD_A_CHILD_EAT_AT_6MONTHS, HOWMANYTIMESFEEDACHILDFROM9TO12MONTHS.

S2B Table: Unadjusted regression analysis showing the association between maternal employment and child dietary diversity

Variables in the Equation

		B	S.E.	Wald	df	Sig.	Exp(B)	95% C.I.for EXP(B)	
								Lower	Upper
Step 1[a]	employment_REGRESSION(1)	.865	.156	30.765	1	.000	2.375	1.750	3.225
	Constant	.460	.074	38.828	1	.000	1.583		

a. Variable(s) entered on step 1: employment_REGRESSION.

S2C Table: Unadjusted regression analysis showing the association between infant resident and dietary diversity.

Variables in the Equation

		B	S.E.	Wald	df	Sig.	Exp(B)	95% C.I.for EXP(B)	
								Lower	Upper
Step 1[a]	resident_REGRESSION			67.033	3	.000			
	resident_REGRESSION(1)	1.188	.200	35.324	1	.000	3.279	2.216	4.851
	resident_REGRESSION(2)	1.055	.168	39.648	1	.000	2.873	2.068	3.989
	resident_REGRESSION(3)	20.886	40192.970	.000	1	1.000	1177104800.634	.000	.
	Constant	.317	.063	25.219	1	.000	1.372		

a. Variable(s) entered on step 1: resident REGRESSION.

S2D Table: Unadjusted regression analysis showing the association between infant age and dietary diversity

Variables in the Equation

		B	S.E.	Wald	df	Sig.	Exp(B)	95% C.I.for EXP(B)	
								Lower	Upper
Step 1[a]	Childs_age(1)	.943	.112	71.282	1	.000	2.568	2.063	3.197
	Constant	.101	.079	1.609	1	.205	1.106		

a. Variable(s) entered on step 1: Childs age.

S2E Table: Means and standard deviations of child and maternal age, nutrition knowledge.

One-Sample Statistics

	N	Mean	Std. Deviation	Std. Error Mean
age of target child	1503	11.03	3.306	.085
maternal age	1368	30.16	11.977	.324
TOTALKNOWLEDGESCORE	1503	7.00	1.497	.039
total_DDscore	1477	7.04	3.830	.100

S2F Table: Proportion of dietary diversity among infants

		Frequency	Percent	Valid Percent	Cumulative Percent
Valid	Above 9 DDS	521	34.7	35.3	35.3
	Below 9 DDS	956	63.6	64.7	100.0
	Total	1477	98.3	100.0	
Missing	System	26	1.7		
Total		1503	100.0		
