# Supplementary material for: Factors associated with food consumption and dietary diversity among infants aged 6–18 months in Ashanti Region, Ghana
Source: PLoS One. 2023 Nov 30;18(11):e0294864. doi: 10.1371/journal.pone.0294864 (PMC10688859; doi:10.1371/journal.pone.0294864)
Supplement: Attachment — Submitted filename: PONE-D-23-17357_R2.pdf [file pone.0294864.s006.pdf]

PLOS ONE

FACTORS ASSOCIATED WITH FOOD CONSUMPTION AND DIETARY DIVERSITY AMONG INFANTS AGED 6-18 MONTHS IN ASHANTI REGION, GHANA

--Manuscript Draft--

**Manuscript Number:**	PONE-D-23-17357R2
**Article Type:**	Research Article
**Full Title:**	FACTORS ASSOCIATED WITH FOOD CONSUMPTION AND DIETARY DIVERSITY AMONG INFANTS AGED 6-18 MONTHS IN ASHANTI REGION, GHANA
**Short Title:**	Food consumption and dietary diversity indicators among infants in Ghana.
**Corresponding Author:**	Godwin Opoku Agyemang<br>Kwame Nkrumah University of Science and Technology College of Science<br>Kumasi, None GHANA
**Keywords:**	Dietary diversity, food consumption, infants, complementary feeding practices
**Abstract:**	Background<br>Studies have reported a poor diet quality among children in Ghana and other developing countries. Inadequate dietary diversity among children may lead to deficiencies in micronutrient intake necessary for growth and other cognitive functions. Understanding factors associated with infants’ diverse diets is a key step to promoting adequate infant and young child nutrition. This study sought to determine the factors associated with food consumption and dietary diversity (DD) among infants.<br>Methods<br>In this cross-sectional study among 1503 mothers-infant (aged 6–18 months) pairs from rural, urban, and peri-urban districts of Ashanti Region, factors associated with food consumption and DD were evaluated. The FAO 18-food group DD questionnaire was used to determine previous food group intake, while a structured questionnaire was used to capture data on the mother's socio-demographic parameters and child morbidity. Data were analyzed using descriptive statistics, the Chi-square test, and binary logistic regression to compare mothers and infants who had adequate and inadequate DDS of 9 out of 18 food groups and the predictors of dietary diversity.<br>Results:<br>About 64.7% of the infants did not meet the minimum dietary diversity. Over two-third (77.4%) of the children consumed maize porridge the previous day. Foods which were less consumed included vegetables (35%), animal milk (38.9%), and meat (organ 14%, any meat 26%). The mean food group intake from 18 food groups was 7.0, and the majority (64.7%) did not meet the recommended 9 food group intake. Significantly more younger children (6-11 months) (74%, p<0.001) compared with older children (12–18 months) (52.5%) did not meet the minimum DDS. Also compared with the older children, the younger ones had above two times increased odds of inadequate DD (OR = 2.5, p<0.001, 95% CI = 1.4–4.4). When controlled for gender, children from peri-urban areas (OR = 5.2, p= 0.260, 95% CI=0.2 – 93.2) and rural areas (OR= 1.8, p= 0.650, 95% CI=0.2 – 9.3) had increased odds of lower DD than urban children. Children of unemployed caregivers had an increased odds of low DD (OR=2.3 p<0.001, 95% CI 1.7-3.2) compared with children of employed caregivers. Finally, children of caregivers with better nutrition knowledge (nine correct answers from 12 questions) had lower odds of having lower dietary diversity (adjusted OR= 0.9, p=0.85, 95%CI = 0.5- 1.6) than those with less knowledge.<br>Conclusions:<br>Low DD was common among infants and associated with infants age, caregivers’ areas of residence, employment status, and level of nutrition education. Children who did not meet the minimum dietary diversity were not fed particular foods such as vegetables, animal milk, and organ meat.
**Order of Authors:**	Godwin Opoku Agyemang
	Samuel Selorm Attu
	Reginald Adjetey Annan
	Satoru Okonogi

Powered by Editorial Manager® and ProduXion Manager® from Aries Systems Corporation

	Takeshi Sakurai
	Odeafo Asamoah-Boakye
**Opposed Reviewers:**	Sara Abdelsalam<br>The British University in Egypt<br><br>The area of speciality is different from my study.
	Saeed Ahmed<br>Rutland Regional Medical Center<br><br>The area of speciality is different from my study
	Clifford Afoakwah<br>Griffith University<br><br>His area of speciality is different from my study.
**Response to Reviewers:**	Please all comments raised by the editor and reviewer have been addressed accordingly and attached as 'Response to Reviewers'.
**Additional Information:**	
**Question**	**Response**
**Financial Disclosure**<br><br>Enter a financial disclosure statement that describes the sources of funding for the work included in this submission. Review the submission guidelines for detailed requirements. View published research articles from *PLOS ONE* for specific examples.<br><br>This statement is required for submission and **will appear in the published article** if the submission is accepted. Please make sure it is accurate.	The authors received no specific funding for this work.

**Unfunded studies**<br>Enter: *The author(s) received no specific funding for this work.*<br><br>**Funded studies**<br>Enter a statement with the following details:<br>• Initials of the authors who received each award<br>• Grant numbers awarded to each author<br>• The full name of each funder<br>• URL of each funder website<br>• Did the sponsors or funders play any role in the study design, data collection and analysis, decision to publish, or preparation of the manuscript?<br>• **NO** - Include this sentence at the end of your statement: *The funders had no role in study design, data collection and analysis, decision to publish, or preparation of the manuscript.*<br>• **YES** - Specify the role(s) played.<br><br>* typeset	
**Competing Interests**<br><br>Use the instructions below to enter a competing interest statement for this submission. On behalf of all authors, disclose any competing interests that could be perceived to bias this work—acknowledging all financial support and any other relevant financial or non-financial competing interests.<br><br>This statement is required for submission and **will appear in the published article** if the submission is accepted. Please make sure it is accurate and that any funding sources listed in your Funding Information later in the submission form are also declared in your Financial Disclosure statement.<br><br>View published research articles from *PLOS ONE* for specific examples.	The authors have declared that no competing interests exist.

Powered by Editorial Manager® and ProduXion Manager® from Aries Systems Corporation

**NO authors have competing interests**<br><br>Enter: *The authors have declared that no competing interests exist.*<br><br>**Authors with competing interests**<br><br>Enter competing interest details beginning with this statement:<br><br>*I have read the journal's policy and the authors of this manuscript have the following competing interests: [insert competing interests here]*<br><br>* typeset	
**Ethics Statement**<br><br>Enter an ethics statement for this submission. This statement is required if the study involved:<br><br>• Human participants<br>• Human specimens or tissue<br>• Vertebrate animals or cephalopods<br>• Vertebrate embryos or tissues<br>• Field research<br><br>Write "N/A" if the submission does not require an ethics statement.<br><br>General guidance is provided below. Consult the submission guidelines for detailed instructions. **Make sure that all information entered here is included in the Methods section of the manuscript.**	A general ethical approval was obtained for the larger study from the Committee on Human Research Publication and Ethics (CHRPE), School of Medical Science, Kwame Nkrumah University of Science and Technology, and Health Director of Health Services at Asokore Mampong (reference: CHRPE/AP/098/19). Ethical approval was sought for the larger study that the current study is based on. All caregivers gave written consent before included in this study.

**Format for specific study types**<br><br>**Human Subject Research (involving human participants and/or tissue)**<br>• Give the name of the institutional review board or ethics committee that approved the study<br>• Include the approval number and/or a statement indicating approval of this research<br>• Indicate the form of consent obtained (written/oral) or the reason that consent was not obtained (e.g. the data were analyzed anonymously)<br><br>**Animal Research (involving vertebrate animals, embryos or tissues)**<br>• Provide the name of the Institutional Animal Care and Use Committee (IACUC) or other relevant ethics board that reviewed the study protocol, and indicate whether they approved this research or granted a formal waiver of ethical approval<br>• Include an approval number if one was obtained<br>• If the study involved *non-human primates*, add *additional details* about animal welfare and steps taken to ameliorate suffering<br>• If anesthesia, euthanasia, or any kind of animal sacrifice is part of the study, include briefly which substances and/or methods were applied<br><br>**Field Research**<br><br>Include the following details if this study involves the collection of plant, animal, or other materials from a natural setting:<br>• Field permit number<br>• Name of the institution or relevant body that granted permission	
**Data Availability**<br><br>Authors are required to make all data underlying the findings described fully available, without restriction, and from the time of publication. PLOS allows rare exceptions to address legal and ethical concerns. See the PLOS Data Policy and FAQ for detailed information.	No - some restrictions will apply

Powered by Editorial Manager® and ProduXion Manager® from Aries Systems Corporation

A Data Availability Statement describing where the data can be found is required at submission. Your answers to this question constitute the Data Availability Statement and **will be published in the article**, if accepted.<br><br>**Important:** Stating 'data available on request from the author' is not sufficient. If your data are only available upon request, select 'No' for the first question and explain your exceptional situation in the text box.<br><br>Do the authors confirm that all data underlying the findings described in their manuscript are fully available without restriction?	
**Describe where the data may be found in full sentences. If you are copying our sample text, replace any instances of XXX with the appropriate details.**<br><br>• If the data are **held or will be held in a public repository**, include URLs, accession numbers or DOIs. If this information will only be available after acceptance, indicate this by ticking the box below. For example: *All XXX files are available from the XXX database (accession number(s) XXX, XXX.*).<br>• If the data are all contained **within the manuscript and/or Supporting Information files**, enter the following: *All relevant data are within the manuscript and its Supporting Information files.*<br>• If neither of these applies but you are able to provide **details of access elsewhere**, with or without limitations, please do so. For example:<br><br>*Data cannot be shared publicly because of [XXX]. Data are available from the XXX Institutional Data Access / Ethics Committee (contact via XXX) for researchers who meet the criteria for access to confidential data.*<br><br>*The data underlying the results presented in the study are available from (include the name of the third party*	Data cannot be shared publicly due to the fact that it contains sensitive identifying information. However, data are available from the Committee on Human Research, Publication, and Ethics (CHRPE), the ethics board of the School of Medicine and Dentistry of the Kwame Nkrumah University of Science and Technology (KNUST), and Komfo Anokye Teaching Hospital (KATH), Kumasi, Ghana (email: chrpe.knust.kath@gmail.com) for researchers who meet the criteria for access to confidential data.

and contact information or URL).<br>• This text is appropriate if the data are owned by a third party and authors do not have permission to share the data.<br><br>* typeset	
Additional data availability information:	

Powered by Editorial Manager® and ProduXion Manager® from Aries Systems Corporation

Department of Biochemistry
College of Science, KNUST,
Kumasi, Ghana.

Dr. Emily Chenette
The Editor-in-Chief,
PLOS ONE Journal,

Covering Letter of Manuscript

Dear Editor-in-Chief,

I would like to submit the manuscript entitled '**Factors associated with food consumption and dietary diversity among infants aged 6-18 months in Ashanti Region, Ghana'** by **Godwin Opoku Agyemang*, Samuel Selorm Attu, Reginald Adjetey Annan, Satoru Okonogi, Takeshi Sakura, Odeafo Asamoah-Boakye,** to be considered for publication as research article in Plos One Journal. We believe our findings would be of great interest to the readership of this Journal. Dietary diversity is a measure of food intake quality and that can improve micronutrient intake of children. Therefore understanding factors associated with feeding infants diverse diets is a key step to promoting adequate infant and young child nutrition. However, little is known about the food consumption and dietary diversity of infants aged between 6-18 months. Our findings showed that 64.7% of the 1503 children between 6-18 months had poor quality diet. Also the children food consumption consumption consisted predominantly maize, representing 77.4% of consumed food group. Vegetables, animal milk, organ meat and any other meat were the least consumed food group, representing 35%, 38.9%,14% and 26% respectively. Mean food group intake from 18 food groups list was 7.0, depicting below average mean consumption. Children who had lower proportion of the food group consumption were more likely to have poor diet quality. The factors associated with inadequate dietary diversity were child's age, mother's nutritional knowledge and mother's employment status.

I would want to state that this manuscript is a revised version of a previous manuscript (PONE-D-23-17357R1) which came with minor revisions. I also want to add that all the concerns raised by the academic editors have been addressed. Also track changes version has been attached. We have no conflict of interest in this publication and there was no financial support to influence the outcome of this work. As a corresponding author, I confirmed that the manuscript has been read and approved for submission by all co-authors.

We suggest the following academic editors to review our study; **Subhendu Kumar Acharya, Neeloy Ashraful Alam, Nawaf W. Alruwaili, John W. Apolzan.**

We also request the following as opposing reviewers; **Sara Abdelsalam, Clifford Afoakwah, Saeed Ahmed**.

We look forward to hearing from you at your earliest convenience.

Yours faithfully,

Mr. Godwn Opoku Agyemang (Corresponding Author).

Manuscript Click here to access/download;Manuscript;Manuscript.docx

FACTORS ASSOCIATED WITH FOOD CONSUMPTION AND DIETARY DIVERSITY AMONG INFANTS AGED 6-18 MONTHS IN ASHANTI REGION, GHANA

Godwin Opoku Agyemang[1*], Samuel Selorm Attu[1], Reginald Annan[1], Satoru Okonogi[2], Takeshi Sakura[2], Odeafo Asamoah-Boakye[1]

*Correspondence:godwinopokuagyemang6@gmail.com

[1]Department of Biochemistry and Biotechnology, College of Science, Kwame Nkrumah University of Science and Technology, PMB, Kumasi, Ghana.[2]Department of Agricultural and Resource Economics, the University of Tokyo, Japan.

Abstract

Background

Studies have reported a poor diet quality among children in Ghana and other developing countries. Inadequate dietary diversity among children may lead to deficiencies in micronutrient intake necessary for growth and other cognitive functions. Understanding factors associated with infants' diverse diets is a key step to promoting adequate infant and young child nutrition. This study sought to determine the factors associated with food consumption and dietary diversity (DD) among infants.

Methods

In this cross-sectional study among 1503 mothers-infant (aged 6–18 months) pairs from rural, urban, and peri-urban districts of Ashanti Region, factors associated with food consumption and DD were evaluated. The FAO 18-food group DD questionnaire was used to determine previous food group intake, while a structured questionnaire was used to capture data on the mother's socio-demographic parameters and child morbidity. Data were analyzed using descriptive statistics, the Chi-square test, and binary logistic regression to compare mothers and infants who had adequate and inadequate DDS of 9 out of 18 food groups and the predictors of dietary diversity.

Results:

About 64.7% of the infants did not meet the minimum dietary diversity. Over two-third (77.4%) of the children consumed maize porridge the previous day. Foods which were less consumed included vegetables (35%), animal milk (38.9%), and meat (organ 14%, any meat 26%). The mean food group intake from 18 food groups was 7.0, and the majority (64.7%) did not meet the recommended 9 food group intake. Significantly more younger children (6-11 months) (74%, $p<0.001$) compared with older children (12–18 months) (52.5%) did not meet the minimum DDS. Also compared with the older children, the younger ones had above two times increased odds of inadequate DD (OR = 2.5, $p<0.001$, 95% CI = 1.4–4.4). When controlled for gender, children from peri-urban areas (OR = 5.2, p= 0.260, 95% CI=0.2 – 93.2) and rural areas (OR= 1.8, p= 0.650, 95% CI=0.2 – 9.3) had increased odds of lower DD than urban children. Children of unemployed

caregivers had an increased odds of low DD (OR=2.3 p<0.001, 95% CI 1.7-3.2) compared with children of employed caregivers. Finally, children of caregivers with better nutrition knowledge (nine correct answers from 12 questions) had lower odds of having lower dietary diversity (adjusted OR= 0.9, p=0.85, 95%CI = 0.5- 1.6) than those with less knowledge.

Conclusions:

Low DD was common among infants and associated with infants age, caregivers' areas of residence, employment status, and level of nutrition education. Children who did not meet the minimum dietary diversity were not fed particular foods such as vegetables, animal milk, and organ meat.

Keywords: Dietary diversity, food consumption, infants, complementary feeding practices

1. Introduction

The concept of optimum feeding is a key determinant of growth and development among infants and young children [1]. According to the United Nations Children's Fund, the age of 6–23 months is considered the critical period in the first 1000 days of life because infants transition from feeding on breastmilk only to the inclusion of complementary foods [2]. Suboptimal infant feeding practice is a major public health concern among children, particularly in less developed communities [3]. Among infants globally, poor feeding practices among infant and young children are on the rise [4]. According the World Health Organization (2018), only less than 25% of infants between 6-23 months consume the recommended diverse diet [5]. Several studies have reported a poor dietary diversity among infants in developing countries [4]. A high prevalence of minimum dietary diversity has been reported in South Africa (43.9%), Tanzania (26%), and Ethiopia (23.3%) [6]. A study conducted by Frempong and Annim revealed that 47% of children in Ghana met the minimum dietary diversity [7]. Studies show that complementary foods given to young children in Ghana are mostly foods of plant origin and tend to have reduced amounts of nutrients [8]. Majority of infants in sub-Saharan Africa (SSA) are fed with cereal based supplemental feeds which are mostly nutritionally inadequate to support rapid growth [9].

Dietary diversity plays an important role in the health of infants. The World Health Organization recommends the provision of a diverse diet to ensure adequate intake of energy, vitamins, proteins, fats, and minerals through complementary feeding for infants and young children aged 6 months and above [10]. *"Minimum dietary diversity is defined as consumption of food from four or more out of seven food groups for higher dietary quality and to meet daily energy and nutrient requirements of the seven recommended food groups namely: grains, roots and tubers; legumes and nuts; dairy products; flesh foods (meat, fish, poultry and organ meat), eggs; vitamin-A rich fruits and vegetables; other fruits and vegetables"* Minimum dietary diversity is one of the eight indicators of infant and young child feeding practices as proposed by the World Health Organization [11]. Increased variety of dietary intake is usually proportional to improved diet quality, characterized by more animal-source foods than a less diversified one [12]. Consistent intake of diverse food sources is essential for adequately meeting nutrient demands [13]. The consumption of a diverse diet during infancy is relevant for maintaining proper health, growth and development of infants and children [14].

Inadequate dietary diversity among children leads to deficiencies in micronutrient intake necessary for growth and other cognitive functions [15]. A large population study reported that the majority of children who did not meet the recommended nutrient intake, including folate, vitamin E, zinc, and fiber, performed poorly in a cognitive test [16]. Consumption of inadequate diverse diet among infants may lead to undernutrition, risk of infections and severe illness [17]. Evidence suggests that poor feeding practices, including an insufficient quantity of complementary and poor-quality foods, are linked with childhood malnutrition [14]. According to the UNICEF, 3.1 million children die from undernutrition yearly. Poor feeding practices at the ages of 6–23 months are the leading cause of undernutrition and other infant morbidities in developing countries, including Ghana [18]

Several studies have reported a positive association between dietary diversity and micronutrient intake, employment status, household food security, socioeconomic status and nutrition knowledge [19,20]. Families with higher income status tend to have consume a diverse diet which results in a positive nutritional status of their infants and young children [20]. Another study which accessed the nutrition knowledge and complementary feeding practices among 1400 mothers in the Northern part of Ghana showed a positive relationship between maternal nutrition knowledge and appropriate feeding practices [21]. According to the study, women who had higher nutrition-related knowledge and attitude were almost twice likely to feed their children with appropriate diet. Poor dietary diversity among infants is reported to be associated with child's age, geographical region and cultural traditional barriers. Understanding factors associated with food consumption and dietary diversity among a large population of infants is a critical step which will be necessary in designing appropriate interventions. This will help improve the proportion of children who are fed with diversified diet in sub-Saharan Africa. However, in Ghana and other sub-Saharan Africa countries, fewer studies have focused on the dietary diversity and food consumption of infants aged between 6 and 18 months who have transitioned from breastmilk only to include complementary foods. Also, very few studies in Ghana have reported on factors associated with poor diet quality in infants aged 6 and 18 months. In this study, we used a large population and primary data to report on factors associated with food consumption and dietary diversity in infants aged between 6 and 18 months. Due to the various food variations in the different regions of Ghana, conducting such a study in Ashanti Region, the second most populous region in Ghana with diverse ethnicity and socioeconomic status will provide a general representation of food consumption and dietary diversity among infants and further serve as a basis for policy implementation.

2. Materials and methods

Study design

Data for this manuscript was generated from a larger study entitled "Experimental Evidence of Effectiveness and Efficiency of Market Based Approach to Improvement of Infant Nutrition in Ghana". The study sought to evaluate the effect of KOKOPLUS infant food supplement on children's nutritional status as well as evaluating the effect of information about children's current nutritional status on demand for KOKOPLUS. The study was a community-based non-controlled intervention study among mother-infant (6-18) months old which was conducted in the Child Welfare Clinics (CWC) in Asokore Mampong Municipality and Bosomtwe District of the Ashanti Region of Ghana. Ashanti Region is the second most populous city in Ghana noted for its ethnic

diversity. Also noted for its farming activities, the region has viable lands which support the cultivation of several crops, fruits and vegetables. It is also noted for economic and other trading activities. Therefore, it is believed that with the availability and access to the different food groups in the region, genuine factors associated with dietary diversity will be determined. Also, the presence of different ethnic groups in the region could be a general representation of the entire country. Asokore Mampong Municipality is one of the newly carved districts and forms part of the region's 43 Metropolitan, Municipal, and District Assemblies (MMDAs). It is an urban community with an estimated population of 372, 222 inhabitants, according to the Population and Housing Census of 2010. The land has an area of 23.9 km^2 and a density of 15,558/km^2. The township of Asokore Mampong can be described as a society of mixed ethnicity. The dominant group is the Akans (40.9%), followed by people from the Northern Region, (36.7%). On the other hand, Bosomtwe District is one of the 43 Districts in the Ashanti Region, located in the Middle Belt Authority of Ghana. In the 2010 census, Bosomtwe District had a population of 93, 910. A majority of the district is made up of Ashanti with smaller proportions of the ethnic group like Ga, Dagombas, and Fante. About 36.3% of the district’s population is employed in agriculture. The study captured a baseline data between April and May, 2019 after which six months monthly follow up was conducted between June and November 2019. An end of intervention was also conducted between November and December, 2019. This manuscript was written from data generated in the baseline study. The baseline captured data on breastfeeding and complementary feeding knowledge and practices, anthropometric data and household socioeconomic status.

Study population and sampling method

A total of 1503 mothers and their indexed infants aged 6–18 months were recruited for the study. A total of 28 CWCs were selected from a total of 40. All mothers whose infants were aged 6–18 months and were attending the selected CWC were recruited during the four weeks of the recruitment period. The mothers were conveniently sampled during their routine visits to the community weighing centers (CWC) in their communities. Mothers who had twin infants and those whose children were outside the age bracket of the study were excluded. The date of birth (day/month/year) of infants, which was recorded in the child’s weight records books, was used to determine the infants ages. A list of mother-infant (6–18 months) pairs from the community weighing centers (CWC) was generated, and every child within the selected age range was eligible for inclusion.

Independent variable:

Data was collected using digital tablets on which the questionnaire had been uploaded to the SurveyBe software application. Data was captured in four parts, including participants' socio-demographic characteristics, maternal nutrition knowledge on breastfeeding, and complementary feeding practices. The age, education, occupation, ethnicity, and religion of the mothers were assessed under the socio-demographic section. The past and current feeding practices of participants were assessed based on the WHO recommendations on infant and young child feeding practices (IYCF). The complementary feeding practices, such as timely initiation of complementary foods, number of complementary foods, food consistency, meal frequency, dietary diversity, use of micronutrient supplements, and feeding behavior during illness and recovery,

were assessed. The WHO's key recommendations for breastfeeding and complementary feeding were used in the assessment of maternal nutrition knowledge and practices. A total of thirteen (13) questions were adopted. Questions were adapted from the WHO indicators for infant feeding. A correct score of "1" was given for a single question, while a "0" was given for an incorrect answer. The total marks were obtained by the summation of the "1" scored for all questions. A participant who scored more than 9 out of the 13 questions, which is equivalent to 70%, was then classified as having adequate nutrition knowledge on breastfeeding, while others were otherwise classified as having inadequate nutrition knowledge. This grading was in line with a similar study conducted in Ghana by [14]. Respondents were asked when they started giving anything apart from breastmilk to a child. The amount of food given to the child in the next 24 hours was estimated. The consumption of foods from specific food groups was also assessed. Food groups included grains, roots, tubers, dairy, eggs, legumes, nuts, fruits, vegetables, and fleshy foods. This was based on the mother's ability to recall foods given to her child in the past 24 hours.

Dependent Variable

Caregivers were asked to select from a list of 18 food items the number of foods consumed by their children in the past 24 hours [22]. These eighteen (18) foods were captured from the eight food groups proposed by the Food and Agriculture Organization (2010), for screening for the minimum dietary diversity. For each food group consumed by the infant in the past 24 hours before data collection, a score of "1" was given, and "0" was given for non-consumed foods. The dietary diversity score was determined from the sum of all consumed food groups on the 18-food list. We defined the dietary diversity of the children by using a similar definition for the minimum dietary diversity of women (MDD-W). In the MDD-W, 10 food groups are used to determine the dietary diversity of women and pregnant women. Consuming five or more food groups is classified as adequate dietary diversity, while consuming less than five is classified as inadequate dietary diversity. Similarly, this study used an expanded 18-food list. A cut-off of nine or more food groups was used to define 'met dietary diversity, while consuming less than nine was defined as 'not met dietary diversity.

Ethics

A general ethical approval was obtained for the larger study from the Committee on Human Research Publication and Ethics (CHRPE), School of Medical Science, Kwame Nkrumah University of Science and Technology, and Health Director of Health Services at Asokore Mampong (reference: CHRPE/AP/098/19). This current manuscript forms part of the larger study. Ethical approval was given in the name of Miss Nancy Elizabeth Tandoh who was part of the six postgraduate students who worked on different areas of this larger study. Trained enumerators explained the purpose of the study to the mothers with the help of nurses working at the CWCs. After each data collection, the data was well reviewed and screened for completeness by the supervisors and principal investigators. The purpose of the study was clearly explained to the mothers with the help of nurses working at the CWCs upon recruitment. Before conducting the study, participants were given written consent forms to sign following research ethics regulations.

Data analysis

Data were extracted from the SurveyBe application and uploaded onto the Statistical Package for Social Sciences (SPSS) version 25.0 (SPSS IBM Inc., Chicago, USA) for statistical analysis. Microsoft Excel was used to draw a graph of food group consumption against the percentage of children who consumed the various food groups. A Chi square (fisher's exact test) cross-tabulation analysis was conducted to determine the relationship between child food group consumption and dietary diversity. A chi-squared analysis was also conducted to determine the relationship between sociodemographic variables and dietary diversity. One sample T-test was also used to determine the mean, standard deviation, and error mean for the dietary diversity score, maternal age, and the relationship between child morbidity, maternal nutrition knowledge, and dietary diversity was also examined using the Chi square (fisher's exact test) crosstabulation. A binary logistic regression analysis was conducted for all significant variables to determine the predictors of poor child dietary diversity. All tests were 2-tailed, and a p-value of < 0.05 was defined as statistically significant.

3. Results

Figure 1.0; Distribution of food consumption by children in the past 24 hours

Figure 1 shows the percentage distribution of food consumed by the children in the past 24 hours. Among the food groups, the majority of the children (77.4%) consumed porridge. More than half of the children did not consume foods including vegetables, beans, sugary foods, milk, and oils. Meat consumption was very poor among children, as 26% and 74% did not consume organ meat or any other meat.

Figure 2.0: Dietary diversity among infants between 6-18 months

Figure 2 shows the dietary diversity among the infants. From the figure, dietary diversity was poor among the infants as it can be observed that more than half of the infants (64.7%) did not meet the minimum dietary diversity.

Table 1; Relationship between food consumption and dietary diversity of children

Food consumption	Inadequate DD N (%)	adequate DD N (%)	X^2	P-value	Food consumption	Inadequate DD N (%)	adequate DD N (%)	X^2	P-value
**bread, rice, noodles**					**organ meat**				
Yes	564(53.3)	494(46.7)	212.94	<0.001	Yes	44(21.2)	164(78.8)	201.3	<0.001
no	392(93.6)	27(6.4)			no	912(71.9)	357(28.1)		
**cassava, plantain, cocoyam**					**Any meat**				
Yes	129(39.3)	199(60.7)	119.1	<0.001	Yes	109(28.3)	276(71.7)	302.42	<0.001
no	827(72.0)	322(28.0)			No	847(77.6)	245(22.4)		
**Porridge**					**Beans foods**				
Yes	682(59.7)	460(40.3)	55.26	<0.001	Yes	120(28.1)	307(71.9)	352.84	<0.001
no	274(81.8)	61(18.2)			No	836(79.6)	214(20.4)		
**Any oils**					**yogurt or cheese**				
Yes	232(39.4)	357(60.6)	275.46	<0.001	Yes	132(30.6)	299(69.4)	309.94	<0.001
no	724(81.5)	164(18.5)			No	824(78.8)	222(21.2)		
**Sugary foods**					**fruits**				
Yes	266(43.3)	348(56.7)	210.84	<0.001	Yes	321(43.2)	422(56.8)	303.33	<0.001
no	690(80.0)	173(20.0)			No	635(86.5)	99(13.5)		
**Infant formula**					**carrots or sweet potato**				
Yes	295(54.0)	251(46.0)	43.4	<0.001	Yes	31(18.7)	135(81.3)	173.71	<0.001
no	661(71.0)	270(29.0)			No	925(70.6)	386(29.4)		
**Tinned powdered or fresh animal milk**					**Dark green leafy vegetables**				
Yes	254(42.2)	336(57.8)	213.44	<0.001	Yes	337(44.8)	416(55.2)	268.36	<0.001
no	711(79.4)	185(20.6)			No	619(85.5)	105(14.5)		
**Egg**					**ripe mangoes or pawpaw**				
Yes	364(45.8)	431(54.2)	270.5	<0.001	Yes	38(19.0)	162(81.0)	211.84	<0.001
no	592(86.8)	90(13.2)			No	918(71.9)	359(28.1)		
**Fish or dried fish**					**Vegetables**				
Yes	446(49.1)	463(50.9)	253.91	<0.001	Yes	196(37.5)	326(62.5)	261.183	<0.001
no	510(89.8)	58(10.2)			No	760(79.6)	195(20.4)		

Data are reported as frequency (percentage), Chi square (X^2) P-values are reported, P-value is significant at $p<0.05$.

Table 1 shows the relationship between food consumption and the dietary diversity of the children. From the table, children who did not consume some foods did not meet the minimum dietary diversity, and children who consumed most foods the previous day were more likely to meet the minimum dietary diversity. The majority of the children who did not consume foods including dark green vegetables, meat, and milk did not meet the dietary diversity. A higher proportion of children who did not consume fruits (86.5%, $P<0.001$) had inadequate dietary diversity score compared to those who consume adequate fruits. Similar observations can be seen in children who did not consume vegetables (79.6%, $P<0.001$). Supplementary Tables 1 and 2 represent food group consumption by age groups and the dietary diversity of children. A higher proportion of 6-11 months old children who did not consume bread, rice, or noodles (96.1%) did not meet the DD ($p<0.001$) compared with 12-18 months old children who did not consume bread, rice, noodles (13.8%). Also, children between 6–11months who did not consume porridge (90.6%) the previous day did not meet the DD ($p<0.001$) as compared to their counterparts between 12 – 18 months who did not consume porridge (69.9%). This can also be seen in the consumption of infant formula, where the younger group that did not consume this food (82.2%) did not meet the DD ($p<0.001$) as compared to the older group (59.2%). A higher proportion of children aged 6–11 months who did not consume any meat (81.8%) did not meet the DD ($p<0.001$) than children between 12–18 months who did not consume any meat ($p<0.001$).

Table 2: Relationship between the socio-demographic factors and dietary diversity score of infants

Sociodemographic variables	Total N (%)	Inadequate DD (< 9 food groups) N=973 (64.7)	Adequate DD (≥9 food groups) N=530 (35.3%)	X^2	P-value
**Total Sample Size**	N=1503				
**Gender of Child**					
Male	750 (49.9)	489 (66.4)	248 (33.6)	1.7	0.212
Female	753 (50.1)	467 (63.1)	273 (36.9)		
**Child's Age (months)**					
6 – 11	856 (57)	622 (74.0)	219 (26.0)	72.93	<0.001
12 – 18	647 (43)	334 (52.5)	302 (47.5)		
**Mother's Age**					
< 20	634 (46.8)	400 (64.1)	224 (35.9)	0.34	0.841
20 – 29	54 (4)	32 (60.4)	21 (39.6)		
30 – 45	668 (49.2)	423 (64.4)	234 (35.6)		
**Resident**					
Rural	189(12.6)	153 (81.8)	34 (18.2)	70.53	<0.001
Peri-Urban	258 (17.2)	205 (79.8)	52 (20.2)		
Urban	1056 (70.3)	598 (57.9)	435 (42.1)		
**Maternal education**					
Basic	298 (20.7)	188 (64.8)	102 (35.2)	0.87	0.832
JHS	750 (52.1)	487 (65.8)	253 (34.2)		
Secondary	283 (19.7)	183 (66.1)	94 (33.9)		
Tertiary	108 (7.5)	74 (69.8)	32 (30.2)		
**Employment status**					
Employed	789 (71)	469 (61.0)	300 (39.0)	32.71	<0.001
Unemployed	322 (29)	252 (79.0)	67 (21.0)		
**Income status**					
1000 cedis and more	56 (12.5)	38 (67.9)	18 (32.1)	0.83	0.653
500 – 999 cedis	118 (26.3)	70 (60.9)	45 (39.1)		
Less than 500 cedis	274 (61.2)	167 (62.1)	102 (37.9)		
**Variable**	**Mean ±mean error (standard deviation)**				
Child age (months)	11.0 ±3.3 (0.8)				
Maternal age	30.1 ±11.9 (0.3)				
Income in cedis (¢)	609.3 ±0.03 (0.6)				

Frequency (percentage) and mean, standard deviation (SD) standard error means are reported, chi-square(X^2) values are reported, and p-values are significant at $p < 0.05$

Table 2 shows the relationship between the socio-demographic factors and the recommended dietary diversity score of the participants. The table also shows the means of the child's age, mother's age, income, and the mean dietary diversity score. The mean age of the children was 11 months. About 65% of the children did not meet the recommended food group intake. A higher proportion of 6-11 months old children (74.0%) had inadequate DD (p<0.001) compared with 12-18 months old children (52.5%). The majority of the children from rural areas (81.8%) could not meet the DD (p<0.001). A greater proportion of children whose mothers were unemployed had inadequate DD (p<0.001).

Table 3: Child morbidity in the past seven days and dietary diversity status of children

Child morbidity in the past 7 days	Inadequate DD (< 9 food groups)	Adequate DD (≥ 9 food groups)	X^2	P-value
**Running Nose**				
Yes	433 (64.4)	239 (35.6)	0.03	0.871
No	521 (64.9)	282 (35.1)		
**Fever**				
Yes	250 (63.0)	147 (37.0)	0.69	0.425
No	704 (65.3)	374 (34.7)		
**Cough**				
Yes	267 (61.9)	164 (38.1)	1.98	0.168
No	687 (65.8)	357 (34.2)		
**Diarrhoea**				
Yes	190 (66.7)	95 (33.3)	0.61	0.449
No	764 (64.2)	426 (35.8)		
**Vomiting**				
Yes	106 (63.1)	62 (36.9)	0.20	0.668
No	848 (64.9)	459 (35.1)		

Frequency (percentage), chi-square (X^2) P values reported, p-values are significant at $p < 0.05$

Table 3 shows the relationship between child morbidity in the past seven days and the dietary diversity status of infants. From the table, there was no significant difference between child morbidity and dietary diversity score, although most of the children who had no illness for the past seven days recorded a low recommended dietary diversity score. However, children who had diarrhea in the previous week (66.7%) were more likely to have inadequate DD compared with those who had no diarrhea (64.2%). Similarly, children who vomited continuously in the previous week (63.1%) were not likely to consume diverse diet as compared to their counterparts who did not vomit (64.9%).

Table 4: Relationship between maternal nutrition knowledge and dietary diversity status of infants

Mothers' nutrition knowledge	Total for mother's knowledge	Inadequate DD (< 9 food groups)	Adequate DD (9 food groups)	X^2	P-value
Good knowledge		130 (59.9)	87 (40.1)	3.21	0.023
Satisfactory Knowledge		775 (65.3)	412 (34.7)		
Poor knowledge		51 (69.9)	22 (30.1)		
**Mean knowledge score**	**7.0 ± 1.4**				**p<0.001**
When should a woman breastfeed after birth					
Correct		822 (65.4)	435 (34.6)	1.64	0.221
Wrong		134 (60.9)	86 (39.1)		
How long should you exclusively breastfeed without adding any drink or food					
Correct		913 (64.7)	499 (35.3)	0.20	0.630
Wrong		27 (61.4)	17 (38.6)		
How long should you breastfeed before stopping or weaning					
Correct		705 (63.9)	399 (36.1)	1.44	0.231
Wrong		251 (67.3)	122 (32.7)		
When should a woman start to give water after birth					
Wrong		954 (64.7)	521 (35.3)	0.3	0.601
Correct		549 (61.2)	445 (38.8)	0.3	
When should a woman give food after birth?					
Correct		835 (64.8)	454 (35.2)	0.10	0.742
Wrong		117 (63.6)	67 (36.4)		
When should the child start eating family food?					
Correct		551 (61.9)	339 (38.1)	7.66	<0.001
Wrong		386 (69.1)	173 (30.9)		
How much complementary food should one give at 6 months					
Correct		597 (67.9)	282 (32.1)	9.59	<0.001
Wrong		358 (60.1)	238 (39.9)		
How much complementary food should one give a 7 – 8 months					
Correct		260 (62.2)	158 (37.8)	1.62	0.203
Wrong		694 (65.7)	362 (34.3)		
How much complementary food should one give at 9 – 12 months					
Correct		251 (57.8)	183 (42.2)	0.12	0.743
Wrong		297 (56.7)	227 (43.3)		
How many times should you give complementary food at 6 – 8 months					
Correct		683 (64.7)	373 (35.3)	0.00	1
Wrong		271 (64.8)	147 (35.2)		
How many times should you give complementary food at 9 – 12 months					
Correct		104 (55.9)	82 (44.1)	7.11	0.009
Wrong		847 (65.9)	438 (34.1)		
Should the child be fed different kinds of food or the same type					

Correct		924 (64.3)	512 (35.7)	3.81	0.061
Wrong		31 (79.5)	8 (20.5)		

Frequency (percentage), chi-square P values reported, p-values are significant at p < 0.05

Table 4 shows the relationship between nutrition knowledge and the dietary diversity status of the children. The mean nutrition knowledge of the mothers was 7.0 (p<0.001). Children of caregivers who had poor nutrition knowledge (69.9%, P = 0.023) had low dietary diversity scores. Children whose caregivers knew when to provide them with family food (61.9%) had better dietary diversity scores than children whose caregivers did not know when to start giving family food to their children (69.1, p<0.001). Also, caregivers who knew how many times to give complementary food to their children at 9–12 months had their children record better dietary diversity scores (44.1%, 0.00) as compared to their counterparts who did not know (34.1%, p<0.001). However, majority of children of caregivers who knew the quantity of complementary food to give them rather had poor dietary diversity scores (67.9%, p<0.001).

Table 5: Predictors of factors associated with child's dietary diversity

Predictable variable	Adjusted OR (95%CI)	P-value	Unadjusted OR (95%CI)	P-value
**Age**				
12 – 18 months	1		1	
6 – 11 months	2.5 (1.4 – 4.4)	<0.001	2.5 (2.0 - 3.1)	<0.001
**Residence**				
Urban	1		1	
Rural	1.8 (0.1 – 29.1)	0.651	3.2 (2.2 - 4.8)	<0.001
Peri – urban	5.2 (0.2 – 93.2)	0.261	2.8 (2.0 - 3.9)	<0.001
**Employment status**				
Employed	1		1	
Unemployed	1.1 (0.1 – 10.6)	0.925	2.3 (1.7 - 3.2)	<0.001
**Maternal Nutrition knowledge;**				
1. When should a woman give the family food	1.8 (1.0 – 3.2)	0.025	1.3 (1.0 - 1.7)	<0.001
2. how much complementary food should a child eat at 6months	0.9 (0.5 – 1.6)	0.851	1.4 (1.1 - 1.7)	<0.001
3. How many times should you feed a child at 9 months to 12 months	0.9 (0.4 – 2.2)	0.935	1.5 (1.1 - 2.0)	<0.001

OR- Odds ratio, AOR- Adjusted odds ratio, CI- Confidence interval, and p-values are significant at p<0.05, adjusted for gender

Table 5 indicates the predictive factors associated with Child's dietary diversity. Children within 6-11 months (adjusted OR=2.5, p<0.001, 95%CI=1.4 – 4.4) had higher odds of having low dietary diversity compared to the children between 12-18 months. Children who lived in rural (unadjusted OR= 3.2, p<0.001, 95%CI= 2.2-4.8) and peri-urban centers (unadjusted OR= 2.8, p<0.001,

95%CI= 2.0-3.9) had higher odds of having a low dietary diversity as compared to children who lived in urban centers. Children whose mothers were unemployed (unadjusted OR= 2.3, p<0.001, 95%CI= 1.7-3.2) had increased odds of having low dietary diversity as compared to their counterparts whose mothers were employed. Children whose mothers had adequate nutrition knowledge had fewer odds of having lower dietary diversity (Adjusted OR= 0.9, p=0.851, 95%CI = 0.5- 1.6)

4. Discussion

This study identified factors associated with food consumption and dietary diversity among infants in Ghana. Severally implemented policies in Ghana including the National Nutrition Policy has been directed at ensuring the healthy growth and development of infants and young children. However, poor feeding practices are reported among children and young infants in Ghana. From our study, the majority of the children consumed porridge (77.4%) and bread, rice, and noodles (71.6%). A similar study in Ghana reported 81% consumption of bread, noodles, and other grains among children [23]. On the contrary, the intake of milk, vegetables, and meat was very low among the children. Similarly, a study among infants between 6-23 months in rural communities in Ethiopia reported low consumption of animal products including meat and milk [24]. Our study found that more than 6 in 10 (64.7%) children had poor-quality diets. This represents a very low level of dietary diversity among the children. Our findings are in line with a study conducted in Ghana by Bandoh and Kenu, which reported a prevalence of 54% of children not meeting the minimum dietary diversity [25]. A higher prevalence of low dietary diversity was reported among infants between 6-23 months in Tanzania [6]. This indicates that the majority of children in the country are likely to be prone to micronutrient deficiencies. Poor feeding among infants is the cause of infant morbidity and mortality. Therefore, necessary nutrition interventions must be put in place to tackle such issues. A popular approach to curtailing this problem has been the free distribution of nutritionally dense complementary foods or supplements [26].

The findings revealed that some socio-demographic factors were associated with child dietary diversity. Child age was found to be associated with dietary diversity, as 74% of the children who did not meet the minimum dietary diversity were between 6-11 months. According to the study, children in this age group were twice at risk of having a low dietary diversity as compared to their counterparts between 12-18 months. This is consistent with other studies that reported a minimum dietary diversity score among children who were between 6-11 months old in Ghana [27]. The reason is due to the preparedness of older infants to accept food in various forms and their high familiarity with certain foods as compared to younger infants. According to Mura Paroche et al., exposure of an infant to a variety of diets at a young age is efficient in ensuring the liking and consumption of both exposed and new foods [28].

Our study also reported how child residence was a predictor of dietary diversity. About 41% of the children who met the minimum dietary diversity were living in urban centers. Children in rural areas had three times the odds of having a lower dietary diversity than their counterparts in urban centers. Similar findings were seen in a study conducted by [29] among Ghanaian adolescents, where rural dwellers had higher odds of inadequate dietary diversity compared to those in urban centers. A study in Nepal reported a higher prevalence of inadequate dietary diversity among children living in developed areas compared to less developed areas. [30] This can also be seen in

other studies that reported a more diversified food intake among urban residents as compared to rural residents [31, 32]. Urban centers are characterized by food access and high economic status, which may explain the decreased risk of having low dietary diversity as compared to rural areas [33]. Majority of rural areas in Ghana are noted for farming of foodstuffs, fruits and vegetables, however, such cultivation is usually meant for commercial purposes to generate income for the family. Though was not found in the study, certain cultural practices prominent in the rural areas might account for the poor dietary diversity among the children. In certain rural communities, infants are usually not fed with milk with the reason that infants will be spoilt when fed with such non-staple diet [26]. About 37% of pregnant women in a study reported one or more food practices influenced by local beliefs and cultural taboos [34]. Such poor cultural beliefs should not be encouraged since it could hinder the consumption of some micronutrients among infants.

According to our study, maternal employment status is also a predictor of infant dietary diversity. Unemployed women had twice the odds of having a lower dietary diversity score for their children as compared to the other women who were employed. Similar issues were seen in a study conducted by Tampah-Naah, where mothers in Ghana who had challenges accessing complementary food items was due to a lack of income or insufficient financial endowment [35]. In a similar study which reported a low dietary diversity among infants, a higher proportion of mothers could not afford a diverse diet because they had difficulty obtaining a decent income [36]. Interventional policies to engage women in livelihood support, as well as other financial support, are critical to addressing this issue. In 2008, the Livelihood Empowerment Against Poverty was initiated to established in Ghana to improve basic household consumption and nutrition, and increase access to welfare services. Strengthening this policy will provide women access to better livelihood where pregnant women can afford nutrient-dense foods.

According to the study, child morbidity was not a predictor of dietary diversity; however, other studies have reported the contrary. Findings from a study by [37] found that child morbidity was a contributing factor to low dietary diversity. Their study also reported low dietary diversity among children suffering from diarrhea. This is, however, consistent with our study, where children who had diarrhea in the previous week (66.7%) were more likely not to meet the DD compared with those who had no diarrhea (64.2%).

Though certain studies have identified a positive association between maternal education level and child dietary diversity [19,38] our study found no association between maternal educational level and dietary diversity. Similarly, level of education among breastfeeding mothers in India was not associated with breastfeeding practices [39]. However, from our findings, children from mothers who had relatively adequate nutrition knowledge had higher chances of meeting the minimum dietary diversity as compared to the other group. Maternal nutrition knowledge has been established as a predictor of infant feeding practices and nutritional status. A similar observation is seen in a study in Ghana by [40], where households with caregivers who had adequate nutrition knowledge and attitude scores had more diverse diets compared to those with little or no nutrition knowledge. Proper maternal nutrition education interventions are therefore critical to solving such situations. Currently, the Ghana Health Service recommends the intake of a four-star diet for a healthy nutrition. Through the health care systems, pregnant and breastfeeding mothers as part of the antenatal and post-natal services are educated on the consumption of the four-star diet.

Empowering pregnant women with nutrition knowledge will help them make better choices which will eventually ensure a healthy nutrition outcome.

Strengths and limitations of the study

Our study used participants from different socioeconomic setting to determine the factors that were associated with food consumption and dietary diversity among the children as compared to previous studies in Ghana and Sub-Sahara Africa. However, our study was not without limitations. Other factors which can also affect child dietary diversity such as food availability and accessibility and cultural and ethnic factors were not assessed in our study. Moreover, the use of the 24-hour dietary recall is not a true indicator of dietary diversity though it is widely used. However, the 24-hour dietary recall is less subject to recall errors, less cumbersome for the respondent and conforms to the recall time period used in many dietary diversity studies.

Conclusions

The study found that infants had poor-quality diet. The majority of the infants also consumed fewer food groups from the 18-food group list. Significant factors associated with poor dietary diversity were the child's age, maternal employment status, residence, and maternal nutrition knowledge. Our study has shown that the factors associated with poor dietary diversity among the infants were multifactorial ranging from age-specific, occupation, nutritional knowledge of mother and residence of infants. Based on the findings in this study, we suggest that proper maternal nutrition education and feeding practices targeting age-specific needs and community livelihood support systems are necessary to improve dietary diversity of infants.

Data Availability

Data cannot be shared publicly ~~due to the fact that~~ it contains sensitive identifying information. However, data are available from the Committee on Human Research, Publication, and Ethics (CHRPE), the ethics board of the School of Medicine and Dentistry of the Kwame Nkrumah University of Science and Technology (KNUST), and Komfo Anokye Teaching Hospital (KATH), Kumasi, Ghana (email: chrpe.knust.kath@gmail.com) for researchers who meet the criteria for access to confidential data.

Funding

The authors received no specific funding for this study.

Acknowledgment

We thank the directors of health and health workers in the health centers where the study took place and most of all the mothers and infants who participated in the study.

Authors contribution

Conceptualization: Reginald Adjetey Annan, Godwin Opoku Agyemang,

Data curation: Godwin Opoku Agyemang, Reginald Adjetey Annan, Samuel Selorm Attu, Odeafo Asamoah Boakye

Formal analysis: Godwin Opoku Agyemang, Reginald Adjetey Annan, Odeafo Asamoah-Boakye

Fund acquisition: Reginald Adjetey Annan, Godwin Opoku Agyemang

Investigation: Godwin Opoku Agyemang, Reginald Adjetey Annan

Project administration: Reginald Adjetey Annan, Godwin Opoku Agyemang, Samuel Selorm Attu

Resources: Reginald Adjetey Annan, Godwin Opoku Agyemang

Software: Reginald Adjetey Annan, Godwin Opoku Agyemang, Odeafo Asamoah-Boakye

Supervision: Reginald Adjetey Annan, Satoru Okonogi, Takeshi Sakurai

Writing- Review and editing: Reginald Adjetey Annan, Godwin Opoku Agyemang, Odeafo Asamoah-Boakye, Satoru Okonogi, Takeshi Sakurai, Samuel Selorm Attu

REFERENCES

1. Gizaw G, Tesfaye G. Minimum acceptable diet and factor associated with it among infant and young children age 6-23 months in north Shoa, Oromia region, Ethiopia. Int J Homeopathy Nat Med. 2019;5(1):1. doi:10.11648/j.ijhnm.20190501.11

2. United Nations Children's Fund (UNICEF), World Health Organization (WHO) & International Bank for Reconstruction and Development/The World Bank (2020) Levels and Trends in Child Malnutrition: Key Findings of the 2020 Edition of the Joint Child Malnutrition Estimates.
3. Ng CS, Dibley MJ, Agho KE. Complementary feeding indicators and determinants of poor feeding practices in Indonesia: a secondary analysis of 2007 Demographic and Health Survey data. Public health nutrition. 2012 May;15(5):827-39. https://doi.org/10.1017/s1368980011002485
4. Eshete T, Kumera G, Bazezew Y, Mihretie A, Marie T. Determinants of inadequate minimum dietary diversity among children aged 6–23 months in Ethiopia: secondary data analysis from Ethiopian Demographic and Health Survey 2016. Agriculture & Food Security. 2018 Dec;7(1): https://doi.org/10.1186/s40066-018-0219-8

5. WHO. Infant and young child feeding. 2018. Available through https://www.who.int/news-room/fact-sheets/detail/infant-and-young-child-feeding. Retrieved on 21 February 2019.
6. Khamis AG, Mwanri AW, Ntwenya JE, Kreppel K. The influence of dietary diversity on the nutritional status of children between 6 and 23 months of age in Tanzania. BMC pediatrics. 2019 Dec;19(1):1-9. https://doi.org/10.1186/s12887-019-1897-5

7. Frempong RB, Annim, S K: Dietary diversity and child malnutrition in Ghana. *Heliyon*, (2017). *3*(5), e00298. https://doi.org/10.1016/j.heliyon.2017.e00298

8. Nti CA: Dietary diversity is associated with the nutrient intake and nutritional status of children in Ghana. *Asian Journal of Medical Sciences*, (2011).*2*(2), 105-109.

9. Onyango AW. Dietary diversity, child nutrition and health in contemporary African communities. Comparative biochemistry and physiology part A: Molecular & Integrative Physiology. 2003 Sep 1;136(1):61-9. https://doi.org/10.1016/s1095-6433(03)00071-0

10. World Health Organization. (2008). *Indicators for assessing infant and young child feeding practices part 3: country profiles*. World Health Organization.

11. World Health Organization (WHO) (2008) Indicators for Assessing Infant and Young Child Feeding Practices: Part 1.

12. Jalata DD, & Asefa B: Trends and Determinants of Dietary Diversity in Children Aged 6–59 Months in Ethiopia (2022) Analysis of 2005–2016 Demographic and Health Survey. *Current Developments in Nutrition*, *6*(10), nzac135. https://doi.org/10.1093/cdn/nzac135

13. Wiafe MA, Apprey C, Annan RA:.Dietary diversity and nutritional status of adolescents in rural Ghana. *Nutrition and Metabolic Insights*, (2023) *16*, 11786388231158487. https://doi.org/10.1177/11786388231158487

14. Saaka M, Larbi A, Mutaru S, Hoeschle-Zeledon, I: Magnitude and factors associated with appropriate complementary feeding among children 6–23 months in northern Ghana. *BMC Nutrition*, (2016)*2*, 1-8. https://doi.org/10.1186/s40795-015-0037-3

15. Annan RA, Apprey C, Asamoah- Boakye O, Okonogi S, Yamauchi T, Sakurai T: The relationship between dietary micronutrient intake and cognition test performance among school- aged children in government- owned primary schools in Kumasi metropolis, Ghana. *Food Science & Nutrition*, (2019)*7*(9), 3042-3051. https://doi.org/10.1002/fsn3.1162

16. Annan RA, Apprey C, Odeafo AB, Benedicta TD, Sakurai T, Okonogi S. Nutrient intakes and cognitive competence in the context of abstract reasoning of school-age children in the Tamale Metropolis of Ghana. Nutrition & Food Science. 2023 Jan 2;53(1):124-37. https://doi.org/10.1108/NFS-11-2021-0343

17. Sirait AR, Achadi EL. Factors Associated with Minimum Dietary Diversity among Breas tfed Children Aged 6-23 Months in Indonesia (Analysis of Indonesia DHS 2017). Indonesian Journal of Public Health Nutrition. 2020 Oct 30;1(1). http://dx.doi.org/10.7454/ijphn.v1i1.4381

18. Erismann S, Knoblauch AM, Diagbouga S, Odermatt P, Gerold J, Shrestha A, et al. Prevalence and risk factors of undernutrition among schoolchildren in the Plateau Central and Centre-Ouest regions of Burkina Faso. Infect Dis Poverty. 2017; 6(1): 17. https://doi.org/10.1186/s40249-016-0230-x

19. Agrawal S, Kim R, Gausman J, Sharma S, Sankar R, Joe W, Subramanian SV. Socio-economic patterning of food consumption and dietary diversity among Indian children: evidence from NFHS-4. European Journal of Clinical Nutrition. 2019 Oct;73(10):1361-72. https://doi.org/10.1038/s41430-019-0406-0

20. Shariff ZM, Lin KG, Sariman S, Lee HS, Siew CY, Yusof BN, Mun CY, Mohamad M. The relationship between household income and dietary intakes of 1-10 year old urban Malaysian. Nutrition research and practice. 2015 Jun 1;9(3):278-87. https://doi.org/10.4162/nrp.2015.9.3.278

21. Saaka M, Awini S, Nang E. Prevalence and predictors of appropriate complementary feeding practice among mothers with children 6–23 months in Northern Ghana. World Nutrition. 2022 Jun 30;13(2):14-23. https://doi.org/10.26596/wn.202213214-23

22. Food Agriculture Organization (FAO); Guidelines for measuring household and individual dietary diversity; 2010

23. Anane, I., Nie, F., & Huang, J. (2021). Socioeconomic and geographic pattern of food consumption and dietary diversity among children aged 6–23 months old in Ghana. *Nutrients*, *13*(2), 603. https://doi.org/10.3390/nu13020603

24. Haileselassie M, Redae G, Berhe G, Henry CJ, Nickerson MT, Tyler B, Mulugeta A. Why are animal source foods rarely consumed by 6-23 months old children in rural communities of Northern Ethiopia? A qualitative study. PloS one. 2020 Jan 8;15(1):e0225707. https://doi.org/10.1371/journal.pone.0225707

25. Bandoh DA, Kenu E. Dietary diversity and nutritional adequacy of under-fives in a fishing community in the central region of Ghana. BMC Nutrition. 2017 Dec;3:1-6. https://doi.org/10.1186/s40795-016-0120-4

26. Okonogi S, Annan R, Sakurai T. Improving Infant Nutrition through the Market: Experimental Evidence in Ghana. Available at SSRN 3771891. 2021 Jul 8. https://dx.doi.org/10.2139/ssrn.3771891

27. Issaka AI, Agho KE, Burns P, Page A, Dibley MJ. Determinants of inadequate complementary feeding practices among children aged 6–23 months in Ghana. Public health nutrition. 2015 Mar;18(4):669-78. https://doi.org/10.1017/s1368980014000834
28. Mura Paroche M, Caton SJ, Vereijken CM, Weenen H, Houston-Price C. How infants and young children learn about food: A systematic review. Frontiers in Psychology. 2017 Jul 25;8:1046. https://doi.org/10.3389/fpsyg.2017.01046
29. Gyimah LA, Annan RA, Apprey C, Edusei A, Aduku LN, Asamoah-Boakye O, Azanu W, Lutterodt H. Dietary diversity and its correlates among pregnant adolescent girls in Ghana. PLoS One. 2021 Mar 8;16(3):e0247979. https://doi.org/10.1371/journal.pone.0247979
30. Baek Y, Chitekwe S. Sociodemographic factors associated with inadequate food group consumption and dietary diversity among infants and young children in Nepal. PloS one. 2019 Mar 11;14(3):e0213610. https://doi.org/10.1371/journal.pone.0213610
31. Paramashanti BA, Huda TM, Alam A, Dibley MJ. Trends and determinants of minimum dietary diversity among children aged 6–23 months: a pooled analysis of Indonesia demographic and health surveys from 2007 to 2017. Public Health Nutrition. 2022 Jul;25(7):1956-67. https://doi.org/10.1017/s1368980021004559
32. Amugsi DA, Mittelmark MB, Lartey A. Dietary diversity is a predictor of acute malnutrition in rural but not in urban settings: evidence from Ghana. Child Care Practices, Resources for Care, and Nutritional Outcomes in Ghana: Findings from Demographic and Health Surveys. 2014 Jun 5.
33. Wang H, Liu C, Fan H, Tian X. Rising food accessibility contributed to the increasing dietary diversity in rural and urban China. Asia Pacific journal of clinical nutrition. 2017 Jan;26(4):738-47. https://doi.org/10.6133/apjcn.052016.03
34. Chakona G, Shackleton C. Food taboos and cultural beliefs influence food choice and dietary preferences among pregnant women in the Eastern Cape, South Africa. Nutrients. 2019 Nov 5;11(11):2668. https://doi.org/10.3390/nu11112668
35. Tampah-Naah AM, Kumi-Kyereme A, Amo-Adjei J. Maternal challenges of exclusive breastfeeding and complementary feeding in Ghana. PloS one. 2019 May 2;14(5):e0215285. https://doi.org/10.1371/journal.pone.0215285

36. Chakona G. Social circumstances and cultural beliefs influence maternal nutrition, breastfeeding and child feeding practices in South Africa. Nutrition Journal. 2020 Dec;19(1):1-5. https://doi.org/10.1186/s12937-020-00566-4
37. Rah JH, Akhter N, Semba RD, De Pee S, Bloem MW, Campbell AA, Moench-Pfanner R, Sun K, Badham J, Kraemer K. Low dietary diversity is a predictor of child stunting in rural Bangladesh. European journal of clinical nutrition. 2010 Dec;64(12):1393-8. https://doi.org/10.1038/ejcn.2010.171
38. Sekartaji R, Suza DE, Fauziningtyas R, Almutairi WM, Susanti IA, Astutik E, Efendi F. Dietary diversity and associated factors among children aged 6–23 months in Indonesia. Journal of Pediatric Nursing. 2021 Jan 1;56:30-4. https://doi.org/10.1016/j.pedn.2020.10.006
39. Ghosh P, Rohatgi P, Bose K. Determinants of time-trends in exclusivity and continuation of breastfeeding in India: An investigation from the National Family Health Survey. Social Science & Medicine. 2022 Jan 1;292:114604. https://doi.org/10.1016/j.socscimed.2021.114604
40. Christian AK, Marquis GS, Colecraft EK, Lartey A, Sakyi-Dawson O, Ahunu BK, Butler LM. Caregivers' nutrition knowledge and attitudes are associated with household food diversity and children's animal source food intake across different agro-ecological zones in Ghana. British Journal of Nutrition. 2016 Jan;115(2):351-60. https://doi.org/10.1017/s0007114515004468

Supplementary Table 1: Food group consumption by age groups and dietary diversity of child

Age group (Months)	Food consumption		Dietary diversity score		X^2	P-value
			**Not met DDS (< 9 food groups)**	**met DDS (≥ 9 food groups)**		
Bread, rice, noodles						
6 – 11	Yes		324 (61.0)	302 (39.0)	212.94	<0.001
	No		298 (96.1)	12 (3.9)		
12 – 18	Yes		240 (45.5)	287 (54.5)		
	No		15 (13.8)	94 (86.2)		
Cassava, Plantain, cocoyam						
6 – 11	Yes		75 (58.1)	54 (41.9)	119.11	<0.001
	No		568 (79.8)	144 (20.2)		
12 – 18	Yes		75 (37.7)	128 (62.3)		
	No		259 (59.3)	178 (40.7)		
Porridge						
6 – 11	Yes		448 (69.0)	201 (31.0)	55.26	<0.001
	No		174 (90.6)	18 (9.4)		
12 – 18	Yes		234 (47.5)	259 (52.5)		
	No		100 (69.9)	43 (30.1)		
Any oils (margarine, butter, etc						
6 – 11	Yes		137 (50.2)	136 (49.8)	275.46	<0.001
	No		485 (85.4)	83 (14.6)		
12 – 18	Yes		95 (30.1)	221 (69.9)		
	No		239 (74.7)	81 (25.3)		
Sugary foods (sweet, chocolate)						
6 – 11	Yes		158 (54.5)	132 (45.5)	210.84	<0.001
	No		464 (84.2)	87 (15.8)		
12 – 18	Yes		108 (33.3)	216 (66.7)		
	No		226 (72.4)	86 (27.6)		
Infant formula						
6 – 11	Yes		229 (63.1)	134 (36.9)	43.40	<0.001
	No		393 (82.2)	85 (17.8)		
12 – 18	Yes		66 (36.1)	117 (63.9)		
	No		268 (59.2)	185 (40.8)		
Tinned powdered or fresh animal milk						
6 – 11	Yes		152 (54.1)	129 (45.9)	213.44	<0.001
	No		470 (83.9)	90 (16.1)		
12 – 18	Yes		334 (52.5)	302 (47.5)		
	No		241 (71.7)	95 (28.3)		
Eggs						
6 – 11	Yes		194 (54.3)	163 (45.7)	270.50	<0.001
	No		428 (88.4)	56 (11.6)		

12 – 18	Yes		170 (38.8)	268 (61.2)		
	No		164 (82.8)	34 (17.2)		
Fresh or dried fish						
6 – 11	Yes		237 (55.5)	190 (44.5)	253.91	<0.001
	No		358 (93.0)	29 (7.0)		
12 – 18	Yes		209 (43.4)	273 (56.6)		
	No		125 (81.2)	29 (18.8)		

Frequency (percentage), Fisher's exact test P values reported, p-values are significant at p < 0.05

Supplementary Table 2: Food group consumption by age groups and dietary diversity of child

Age group (Months)	Food Group	Dietary diversity score		X^2	P-value
		**Not met DDS (< 9 food groups)**	**met DDS (≥ 9 food groups)**		
Organ meat					
6 – 11	Yes	27 (32.5)	56 (67.5)	201.30	<0.001
	No	595 (78.5)	163 (21.5)		
12 – 18	Yes	17 (13.6)	108 (86.4)		
	No	317 (62.0)	194 (38.0)		
Any meat					
6 – 11	Yes	51 (35.7)	92 (64.3)	302.42	<0.001
	No	571 (81.8)	127 (18.2)		
12 – 18	Yes	58 (24.0)	184 (76.0)		
	No	276 (70.1)	118 (29.9)		
Foods made from beans, lentils, etc.					
6 – 11	Yes	76 (36.5)	132 (63.5)	140.82	<0.001
	No	546 (86.3)	87 (13.7)		
12 – 18	Yes	44 (20.1)	175 (79.9)		
	No	290 (69.5)	127 (30.5)		
Yogurt or cheese					
6 – 11	Yes	84 (43.3)	110 (56.7)	309.94	<0.001
	No	538 (83.2)	109 (16.8)		
12 – 18	Yes	48 (20.3)	189 (79.7)		
	No	286 (71.7)	113 (28.3)		
Fruits					
6 – 11	Yes	190 (53.1)	168 (46.9)	303.33	<0.001
	No	432 (89.4)	51 (10.6)		
12 – 18	Yes	131 (34.0)	254 (66.0)		
	No	203 (80.9)	48 (19.1)		

Carrots or sweet potato					
6 – 11	Yes	16 (21.3)	59 (78.7)	173.71	<0.001
	No	606 (79.1)	160 (20.9)		
12 – 18	Yes	15 (16.5)	76 (83.5)		
	No	319 (58.5)	226 (41.5)		
Dark green leafy vegetables					
6 – 11	Yes	206 (53.8)	177 (46.2)	268.36	<0.001
	No	416 (90.8)	42 (9.2)		
12 – 18	Yes	131 (35.4)	239 (64.6)		
	No	203 (76.3)	63 (23.7)		
Ripe mangoes or pawpaw					
6 – 11	Yes	17 (20.7)	65 (79.3)	211.84	<0.001
	No	605 (79.7)	154 (20.3)		
12 – 18	Yes	21 (17.8)	97 (82.2)		
	No	313 (60.4)	205 (39.6)		
Any other fruits and vegetables					
6 – 11	Yes	116 (44.1)	147 (55.9)	81.95	<0.001
	No	506 (87.5)	72 (12.5)		
12 – 18	Yes	80 (30.9)	179 (69.1)		
	No	254 (67.4)	123 (32.6)		

Frequency (percentage), Fisher's exact test P values reported, p-values are significant at $p < 0.05$

Regression Analysis showing the predictors of dietary diversity.

Variables in the Equation

		B	S.E.	Wald	df	Sig.	Exp(B)	95% C.I.for EXP(B)	
								Lower	Upper
Step 1[a]	educational_level	.293	.177	2.750	1	.097	1.340	.948	1.895
	INCOMESTATUS	-.215	.225	.915	1	.339	.807	.519	1.253
	Childs_age(1)	.928	.288	10.375	1	.001	2.529	1.438	4.448
	resident_REGRESSION			1.311	2	.519			
	resident_REGRESSION (1)	.628	1.399	.201	1	.654	1.873	.121	29.070
	resident_REGRESSION (2)	1.652	1.471	1.261	1	.261	5.217	.292	93.254
	employment_REGRESSION(1)	.105	1.155	.008	1	.928	1.110	.115	10.676
	WHEN_GIVE_FAMILY_FOOD_GRADE(1)	.634	.277	5.220	1	.022	1.885	1.094	3.246
	HOW_MUCH_COMPLEMENTARY_FOOD_SHOULD_A_CHILD_EAT_AT_6MONTHS(1)	-.049	.268	.033	1	.855	.952	.563	1.610
	HOWMANYTIMESFEEDACHILDFROM9TO12MONTHS(1)	-.038	.439	.007	1	.931	.963	.407	2.278
	Constant	-.709	.634	1.248	1	.264	.492		

a. Variable(s) entered on step 1: Childs_age, resident_REGRESSION, employment_REGRESSION, WHEN_GIVE_FAMILY_FOOD_GRADE, HOW_MUCH_COMPLEMENTARY_FOOD_SHOULD_A_CHILD_EAT_AT_6MONTHS, HOWMANYTIMESFEEDACHILDFROM9TO12MONTHS.

Variables in the Equation

		B	S.E.	Wald	df	Sig.	Exp(B)	95% C.I.for EXP(B)	
								Lower	Upper
Step 1[a]	employment_REGRESSION (1)	.865	.156	30.765	1	.000	2.375	1.750	3.225
	Constant	.460	.074	38.828	1	.000	1.583		

a. Variable(s) entered on step 1: employment_REGRESSION.

Variables in the Equation

		B	S.E.	Wald	df	Sig.	Exp(B)	95% C.I.for EXP(B)	
								Lower	Upper
Step 1[a]	resident_REGRESSION			67.033	3	.000			
	resident_REGRESSION(1)	1.188	.200	35.324	1	.000	3.279	2.216	4.851
	resident_REGRESSION(2)	1.055	.168	39.648	1	.000	2.873	2.068	3.989
	resident_REGRESSION(3)	20.886	40192.970	.000	1	1.000	1177104800.634	.000	.
	Constant	.317	.063	25.219	1	.000	1.372		

a. Variable(s) entered on step 1: resident_REGRESSION.

Variables in the Equation

		B	S.E.	Wald	df	Sig.	Exp(B)	95% C.I.for EXP(B)	
								Lower	Upper
Step 1[a]	Childs_age(1)	.943	.112	71.282	1	.000	2.568	2.063	3.197
	Constant	.101	.079	1.609	1	.205	1.106		

a. Variable(s) entered on step 1: Childs_age.

One-Sample Statistics

	N	Mean	Std. Deviation	Std. Error Mean
age of target child	1503	11.03	3.306	.085
maternal age	1368	30.16	11.977	.324
TOTALKNOWLEDGESCORE	1503	7.00	1.497	.039
total_DDscore	1477	7.04	3.830	.100

Mean_Dietary diversity score

		Frequency	Percent	Valid Percent	Cumulative Percent
Valid	Above 9 DDS	521	34.7	35.3	35.3
	Below 9 DDS	956	63.6	64.7	100.0
	Total	1477	98.3	100.0	
Missing	System	26	1.7		
Total		1503	100.0		

Figure 1 Click here to access/download;Figure;figure 1.tif

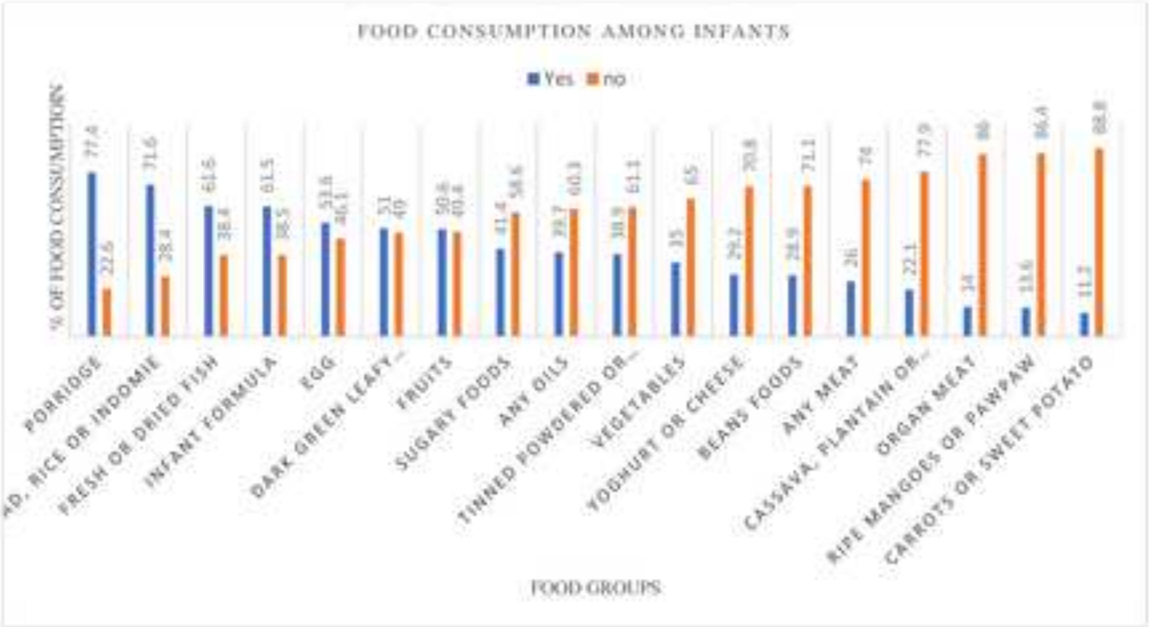

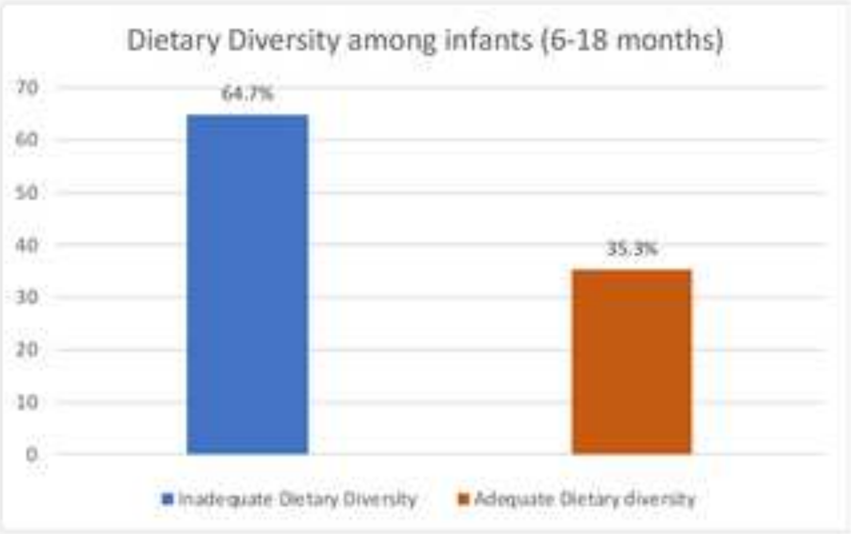
Dietary Diversity among infants (6-18 months)
70
60
50
40
30
20
10
0
64.7%
35.3%
Inadequate Dietary Diversity
Adequate Dietary diversity

Click here to access/download
Other
PLOSOne-Clinical-Studies-Checklist.docx

Click here to access/download
Other
ETHICAL APPROVAL LETTER (2019) KOKOPLUS PROJECT.pdf

Click here to access/download
Other
STROBE-checklist-v4-combined-PlosMedicine.docx

FACTORS ASSOCIATED WITH FOOD CONSUMPTION AND DIETARY DIVERSITY AMONG INFANTS AGED 6-18 MONTHS IN ASHANTI REGION, GHANA

Godwin Opoku Agyemang[1*], Samuel Selorm Attu[1], Reginald Annan[1], Satoru Okonogi[2], Takeshi Sakura[2], Odeafo Asamoah-Boakye[1]

*Correspondence:godwinopokuagyemang6@gmail.com

[1]Department of Biochemistry and Biotechnology, College of Science, Kwame Nkrumah University of Science and Technology, PMB, Kumasi, Ghana.[2]Department of Agricultural and Resource Economics, the University of Tokyo, Japan.

Abstract

Background

Studies have reported a poor diet quality among children in Ghana and other developing countries. Inadequate dietary diversity among children may lead to deficiencies in micronutrient intake necessary for growth and other cognitive functions. Understanding factors associated with infants' diverse diets is a key step to promoting adequate infant and young child nutrition. This study sought to determine the factors associated with food consumption and dietary diversity (DD) among infants.

Methods

In this cross-sectional study among 1503 mothers-infant (aged 6–18 months) pairs from rural, urban, and peri-urban districts of Ashanti Region, factors associated with food consumption and DD were evaluated. The FAO 18-food group DD questionnaire was used to determine previous food group intake, while a structured questionnaire was used to capture data on the mother's socio-demographic parameters and child morbidity. Data were analyzed using descriptive statistics, the Chi-square test, and binary logistic regression to compare mothers and infants who had adequate and inadequate DDS of 9 out of 18 food groups and the predictors of dietary diversity.

Results:

About 64.7% of the infants did not meet the minimum dietary diversity. Over two-third (77.4%) of the children consumed maize porridge the previous day. Foods which were less consumed included vegetables (35%), animal milk (38.9%), and meat (organ 14%, any meat 26%). The mean food group intake from 18 food groups was 7.0, and the majority (64.7%) did not meet the recommended 9 food group intake. Significantly more younger children (6-11 months) (74%, $p<0.001$) compared with older children (12–18 months) (52.5%) did not meet the minimum DDS. Also compared with the older children, the younger ones had above two times increased odds of inadequate DD (OR = 2.5, $p<0.001$, 95% CI = 1.4–4.4). When controlled for gender, children from peri-urban areas (OR = 5.2, p= 0.260, 95% CI=0.2 – 93.2) and rural areas (OR= 1.8, p= 0.650, 95% CI=0.2 – 9.3) had increased odds of lower DD than urban children. Children of unemployed

caregivers had an increased odds of low DD (OR=2.3 p<0.001, 95% CI 1.7-3.2) compared with children of employed caregivers. Finally, children of caregivers with better nutrition knowledge (nine correct answers from 12 questions) had lower odds of having lower dietary diversity (adjusted OR= 0.9, p=0.85, 95%CI = 0.5- 1.6) than those with less knowledge.

Conclusions:

Low DD was common among infants and associated with infants age, caregivers' areas of residence, employment status, and level of nutrition education. Children who did not meet the minimum dietary diversity were not fed particular foods such as vegetables, animal milk, and organ meat.

Keywords: Dietary diversity, food consumption, infants, complementary feeding practices

1. Introduction

The concept of optimum feeding is a key determinant of growth and development among infants and young children [1]. According to the United Nations Children's Fund, the age of 6–23 months is considered the critical period in the first 1000 days of life because infants transition from feeding on breastmilk only to the inclusion of complementary foods [2]. Suboptimal infant feeding practice is a major public health concern among children, particularly in less developed communities [3]. Among infants globally, poor feeding practices among infant and young children are on the rise [4]. According the World Health Organization (2018), only less than 25% of infants between 6-23 months consume the recommended diverse diet [5]. Several studies have reported a poor dietary diversity among infants in developing countries [4]. A high prevalence of minimum dietary diversity has been reported in South Africa (43.9%), Tanzania (26%), and Ethiopia (23.3%) [6]. A study conducted by Frempong and Annim revealed that 47% of children in Ghana met the minimum dietary diversity [7]. Studies show that complementary foods given to young children in Ghana are mostly foods of plant origin and tend to have reduced amounts of nutrients [8]. Majority of infants in sub-Saharan Africa (SSA) are fed with cereal based supplemental feeds which are mostly nutritionally inadequate to support rapid growth [9].

Dietary diversity plays an important role in the health of infants. The World Health Organization recommends the provision of a diverse diet to ensure adequate intake of energy, vitamins, proteins, fats, and minerals through complementary feeding for infants and young children aged 6 months and above [10]. *"Minimum dietary diversity is defined as consumption of food from four or more out of seven food groups for higher dietary quality and to meet daily energy and nutrient requirements of the seven recommended food groups namely: grains, roots and tubers; legumes and nuts; dairy products; flesh foods (meat, fish, poultry and organ meat), eggs; vitamin-A rich fruits and vegetables; other fruits and vegetables"* Minimum dietary diversity is one of the eight indicators of infant and young child feeding practices as proposed by the World Health Organization [11]. Increased variety of dietary intake is usually proportional to improved diet quality, characterized by more animal-source foods than a less diversified one [12]. Consistent intake of diverse food sources is essential for adequately meeting nutrient demands [13]. The consumption of a diverse diet during infancy is relevant for maintaining proper health, growth and development of infants and children [14].

Inadequate dietary diversity among children leads to deficiencies in micronutrient intake necessary for growth and other cognitive functions [15]. A large population study reported that the majority of children who did not meet the recommended nutrient intake, including folate, vitamin E, zinc, and fiber, performed poorly in a cognitive test [16]. Consumption of inadequate diverse diet among infants may lead to undernutrition, risk of infections and severe illness [17]. Evidence suggests that poor feeding practices, including an insufficient quantity of complementary and poor-quality foods, are linked with childhood malnutrition [14]. According to the UNICEF, 3.1 million children die from undernutrition yearly. Poor feeding practices at the ages of 6–23 months are the leading cause of undernutrition and other infant morbidities in developing countries, including Ghana [18]

Several studies have reported a positive association between dietary diversity and micronutrient intake, employment status, household food security, socioeconomic status and nutrition knowledge [19,20]. Families with higher income status tend to have consume a diverse diet which results in a positive nutritional status of their infants and young children [20]. Another study which accessed the nutrition knowledge and complementary feeding practices among 1400 mothers in the Northern part of Ghana showed a positive relationship between maternal nutrition knowledge and appropriate feeding practices [21]. According to the study, women who had higher nutrition-related knowledge and attitude were almost twice likely to feed their children with appropriate diet. Poor dietary diversity among infants is reported to be associated with child's age, geographical region and cultural traditional barriers. Understanding factors associated with food consumption and dietary diversity among a large population of infants is a critical step which will be necessary in designing appropriate interventions. This will help improve the proportion of children who are fed with diversified diet in sub-Saharan Africa. However, in Ghana and other sub-Saharan Africa countries, fewer studies have focused on the dietary diversity and food consumption of infants aged between 6 and 18 months who have transitioned from breastmilk only to include complementary foods. Also, very few studies in Ghana have reported on factors associated with poor diet quality in infants aged 6 and 18 months. In this study, we used a large population and primary data to report on factors associated with food consumption and dietary diversity in infants aged between 6 and 18 months. Due to the various food variations in the different regions of Ghana, conducting such a study in Ashanti Region, the second most populous region in Ghana with diverse ethnicity and socioeconomic status will provide a general representation of food consumption and dietary diversity among infants and further serve as a basis for policy implementation.

2. Materials and methods

Study design

Data for this manuscript was generated from a larger study entitled "Experimental Evidence of Effectiveness and Efficiency of Market Based Approach to Improvement of Infant Nutrition in Ghana". The study sought to evaluate the effect of KOKOPLUS infant food supplement on children's nutritional status as well as evaluating the effect of information about children's current nutritional status on demand for KOKOPLUS. The study was a community-based non-controlled intervention study among mother-infant (6-18) months old which was conducted in the Child Welfare Clinics (CWC) in Asokore Mampong Municipality and Bosomtwe District of the Ashanti Region of Ghana. Ashanti Region is the second most populous city in Ghana noted for its ethnic

diversity. Also noted for its farming activities, the region has viable lands which support the cultivation of several crops, fruits and vegetables. It is also noted for economic and other trading activities. Therefore, it is believed that with the availability and access to the different food groups in the region, genuine factors associated with dietary diversity will be determined. Also, the presence of different ethnic groups in the region could be a general representation of the entire country. Asokore Mampong Municipality is one of the newly carved districts and forms part of the region's 43 Metropolitan, Municipal, and District Assemblies (MMDAs). It is an urban community with an estimated population of 372, 222 inhabitants, according to the Population and Housing Census of 2010. The land has an area of 23.9 km^2 and a density of 15,558/km^2. The township of Asokore Mampong can be described as a society of mixed ethnicity. The dominant group is the Akans (40.9%), followed by people from the Northern Region, (36.7%). On the other hand, Bosomtwe District is one of the 43 Districts in the Ashanti Region, located in the Middle Belt Authority of Ghana. In the 2010 census, Bosomtwe District had a population of 93, 910. A majority of the district is made up of Ashanti with smaller proportions of the ethnic group like Ga, Dagombas, and Fante. About 36.3% of the district's population is employed in agriculture. The study captured a baseline data between April and May, 2019 after which six months monthly follow up was conducted between June and November 2019. An end of intervention was also conducted between November and December, 2019. This manuscript was written from data generated in the baseline study. The baseline captured data on breastfeeding and complementary feeding knowledge and practices, anthropometric data and household socioeconomic status.

Study population and sampling method

A total of 1503 mothers and their indexed infants aged 6–18 months were recruited for the study. A total of 28 CWCs were selected from a total of 40. All mothers whose infants were aged 6–18 months and were attending the selected CWC were recruited during the four weeks of the recruitment period. The mothers were conveniently sampled during their routine visits to the community weighing centers (CWC) in their communities. Mothers who had twin infants and those whose children were outside the age bracket of the study were excluded. The date of birth (day/month/year) of infants, which was recorded in the child's weight records books, was used to determine the infants ages. A list of mother-infant (6–18 months) pairs from the community weighing centers (CWC) was generated, and every child within the selected age range was eligible for inclusion.

Independent variable:

Data was collected using digital tablets on which the questionnaire had been uploaded to the SurveyBe software application. Data was captured in four parts, including participants' socio-demographic characteristics, maternal nutrition knowledge on breastfeeding, and complementary feeding practices. The age, education, occupation, ethnicity, and religion of the mothers were assessed under the socio-demographic section. The past and current feeding practices of participants were assessed based on the WHO recommendations on infant and young child feeding practices (IYCF). The complementary feeding practices, such as timely initiation of complementary foods, number of complementary foods, food consistency, meal frequency, dietary diversity, use of micronutrient supplements, and feeding behavior during illness and recovery,

were assessed. The WHO's key recommendations for breastfeeding and complementary feeding were used in the assessment of maternal nutrition knowledge and practices. A total of thirteen (13) questions were adopted. Questions were adapted from the WHO indicators for infant feeding. A correct score of "1" was given for a single question, while a "0" was given for an incorrect answer. The total marks were obtained by the summation of the "1" scored for all questions. A participant who scored more than 9 out of the 13 questions, which is equivalent to 70%, was then classified as having adequate nutrition knowledge on breastfeeding, while others were otherwise classified as having inadequate nutrition knowledge. This grading was in line with a similar study conducted in Ghana by [14]. Respondents were asked when they started giving anything apart from breastmilk to a child. The amount of food given to the child in the next 24 hours was estimated. The consumption of foods from specific food groups was also assessed. Food groups included grains, roots, tubers, dairy, eggs, legumes, nuts, fruits, vegetables, and fleshy foods. This was based on the mother's ability to recall foods given to her child in the past 24 hours.

Dependent Variable

Caregivers were asked to select from a list of 18 food items the number of foods consumed by their children in the past 24 hours [22]. These eighteen (18) foods were captured from the eight food groups proposed by the Food and Agriculture Organization (2010), for screening for the minimum dietary diversity. For each food group consumed by the infant in the past 24 hours before data collection, a score of "1" was given, and "0" was given for non-consumed foods. The dietary diversity score was determined from the sum of all consumed food groups on the 18-food list. We defined the dietary diversity of the children by using a similar definition for the minimum dietary diversity of women (MDD-W). In the MDD-W, 10 food groups are used to determine the dietary diversity of women and pregnant women. Consuming five or more food groups is classified as adequate dietary diversity, while consuming less than five is classified as inadequate dietary diversity. Similarly, this study used an expanded 18-food list. A cut-off of nine or more food groups was used to define 'met dietary diversity, while consuming less than nine was defined as 'not met dietary diversity.

Ethics

A general ethical approval was obtained for the larger study from the Committee on Human Research Publication and Ethics (CHRPE), School of Medical Science, Kwame Nkrumah University of Science and Technology, and Health Director of Health Services at Asokore Mampong (reference: CHRPE/AP/098/19). This current manuscript forms part of the larger study. Ethical approval was given in the name of Miss Nancy Elizabeth Tandoh who was part of the six postgraduate students who worked on different areas of this larger study. Trained enumerators explained the purpose of the study to the mothers with the help of nurses working at the CWCs. After each data collection, the data was well reviewed and screened for completeness by the supervisors and principal investigators. The purpose of the study was clearly explained to the mothers with the help of nurses working at the CWCs upon recruitment. Before conducting the study, participants were given written consent forms to sign following research ethics regulations.

Data analysis

Data were extracted from the SurveyBe application and uploaded onto the Statistical Package for Social Sciences (SPSS) version 25.0 (SPSS IBM Inc., Chicago, USA) for statistical analysis. Microsoft Excel was used to draw a graph of food group consumption against the percentage of children who consumed the various food groups. A Chi square (fisher's exact test) cross-tabulation analysis was conducted to determine the relationship between child food group consumption and dietary diversity. A chi-squared analysis was also conducted to determine the relationship between sociodemographic variables and dietary diversity. One sample T-test was also used to determine the mean, standard deviation, and error mean for the dietary diversity score, maternal age, and the relationship between child morbidity, maternal nutrition knowledge, and dietary diversity was also examined using the Chi square (fisher's exact test) crosstabulation. A binary logistic regression analysis was conducted for all significant variables to determine the predictors of poor child dietary diversity. All tests were 2-tailed, and a p-value of < 0.05 was defined as statistically significant.

3. Results

Figure 1.0; Distribution of food consumption by children in the past 24 hours

Figure 1 shows the percentage distribution of food consumed by the children in the past 24 hours. Among the food groups, the majority of the children (77.4%) consumed porridge. More than half of the children did not consume foods including vegetables, beans, sugary foods, milk, and oils. Meat consumption was very poor among children, as 26% and 74% did not consume organ meat or any other meat.

Figure 2.0: Dietary diversity among infants between 6-18 months

Figure 2 shows the dietary diversity among the infants. From the figure, dietary diversity was poor among the infants as it can be observed that more than half of the infants (64.7%) did not meet the minimum dietary diversity.

Table 1; Relationship between food consumption and dietary diversity of children

Food consumption	Inadequate DD N (%)	adequate DD N (%)	X^2	P-value	Food consumption	Inadequate DD N (%)	adequate DD N (%)	X^2	P-value
**bread, rice, noodles**					**organ meat**				
Yes	564(53.3)	494(46.7)	212.94	<0.001	Yes	44(21.2)	164(78.8)	201.3	<0.001
no	392(93.6)	27(6.4)			no	912(71.9)	357(28.1)		
**cassava, plantain, cocoyam**					**Any meat**				
Yes	129(39.3)	199(60.7)	119.1	<0.001	Yes	109(28.3)	276(71.7)	302.42	<0.001
no	827(72.0)	322(28.0)			No	847(77.6)	245(22.4)		
**Porridge**					**Beans foods**				
Yes	682(59.7)	460(40.3)	55.26	<0.001	Yes	120(28.1)	307(71.9)	352.84	<0.001
no	274(81.8)	61(18.2)			No	836(79.6)	214(20.4)		
**Any oils**					**yogurt or cheese**				
Yes	232(39.4)	357(60.6)	275.46	<0.001	Yes	132(30.6)	299(69.4)	309.94	<0.001
no	724(81.5)	164(18.5)			No	824(78.8)	222(21.2)		
**Sugary foods**					**fruits**				
Yes	266(43.3)	348(56.7)	210.84	<0.001	Yes	321(43.2)	422(56.8)	303.33	<0.001
no	690(80.0)	173(20.0)			No	635(86.5)	99(13.5)		
**Infant formula**					**carrots or sweet potato**				
Yes	295(54.0)	251(46.0)	43.4	<0.001	Yes	31(18.7)	135(81.3)	173.71	<0.001
no	661(71.0)	270(29.0)			No	925(70.6)	386(29.4)		
**Tinned powdered or fresh animal milk**					**Dark green leafy vegetables**				
Yes	254(42.2)	336(57.8)	213.44	<0.001	Yes	337(44.8)	416(55.2)	268.36	<0.001
no	711(79.4)	185(20.6)			No	619(85.5)	105(14.5)		
**Egg**					**ripe mangoes or pawpaw**				
Yes	364(45.8)	431(54.2)	270.5	<0.001	Yes	38(19.0)	162(81.0)	211.84	<0.001
no	592(86.8)	90(13.2)			No	918(71.9)	359(28.1)		
**Fish or dried fish**					**Vegetables**				
Yes	446(49.1)	463(50.9)	253.91	<0.001	Yes	196(37.5)	326(62.5)	261.183	<0.001
no	510(89.8)	58(10.2)			No	760(79.6)	195(20.4)		

Data are reported as frequency (percentage), Chi square (X^2) P-values are reported, P-value is significant at $p<0.05$.

Table 1 shows the relationship between food consumption and the dietary diversity of the children. From the table, children who did not consume some foods did not meet the minimum dietary diversity, and children who consumed most foods the previous day were more likely to meet the minimum dietary diversity. The majority of the children who did not consume foods including dark green vegetables, meat, and milk did not meet the dietary diversity. A higher proportion of children who did not consume fruits (86.5%, $P<0.001$) had inadequate dietary diversity score compared to those who consume adequate fruits. Similar observations can be seen in children who did not consume vegetables (79.6%, $P<0.001$). Supplementary Tables 1 and 2 represent food group consumption by age groups and the dietary diversity of children. A higher proportion of 6-11 months old children who did not consume bread, rice, or noodles (96.1%) did not meet the DD ($p<0.001$) compared with 12-18 months old children who did not consume bread, rice, noodles (13.8%). Also, children between 6–11months who did not consume porridge (90.6%) the previous day did not meet the DD ($p<0.001$) as compared to their counterparts between 12 – 18 months who did not consume porridge (69.9%). This can also be seen in the consumption of infant formula, where the younger group that did not consume this food (82.2%) did not meet the DD ($p<0.001$) as compared to the older group (59.2%). A higher proportion of children aged 6–11 months who did not consume any meat (81.8%) did not meet the DD ($p<0.001$) than children between 12–18 months who did not consume any meat ($p<0.001$).

Table 2: Relationship between the socio-demographic factors and dietary diversity score of infants

Sociodemographic variables	Total N (%)	Inadequate DD (< 9 food groups) N=973 (64.7)	Adequate DD (≥9 food groups) N=530 (35.3%)	X²	P-value
**Total Sample Size**	N=1503				
**Gender of Child**					
Male	750 (49.9)	489 (66.4)	248 (33.6)	1.7	0.212
Female	753 (50.1)	467 (63.1)	273 (36.9)		
**Child's Age (months)**					
6 – 11	856 (57)	622 (74.0)	219 (26.0)	72.93	<0.001
12 – 18	647 (43)	334 (52.5)	302 (47.5)		
**Mother's Age**					
< 20	634 (46.8)	400 (64.1)	224 (35.9)	0.34	0.841
20 – 29	54 (4)	32 (60.4)	21 (39.6)		
30 – 45	668 (49.2)	423 (64.4)	234 (35.6)		
**Resident**					
Rural	189(12.6)	153 (81.8)	34 (18.2)	70.53	<0.001
Peri-Urban	258 (17.2)	205 (79.8)	52 (20.2)		
Urban	1056 (70.3)	598 (57.9)	435 (42.1)		
**Maternal education**					
Basic	298 (20.7)	188 (64.8)	102 (35.2)	0.87	0.832
JHS	750 (52.1)	487 (65.8)	253 (34.2)		
Secondary	283 (19.7)	183 (66.1)	94 (33.9)		
Tertiary	108 (7.5)	74 (69.8)	32 (30.2)		
**Employment status**					
Employed	789 (71)	469 (61.0)	300 (39.0)	32.71	<0.001
Unemployed	322 (29)	252 (79.0)	67 (21.0)		
**Income status**					
1000 cedis and more	56 (12.5)	38 (67.9)	18 (32.1)	0.83	0.653
500 – 999 cedis	118 (26.3)	70 (60.9)	45 (39.1)		
Less than 500 cedis	274 (61.2)	167 (62.1)	102 (37.9)		
**Variable**	**Mean ±mean error (standard deviation)**				
Child age (months)	11.0 ±3.3 (0.8)				
Maternal age	30.1 ±11.9 (0.3)				
Income in cedis (¢)	609.3 ±0.03 (0.6)				

Frequency (percentage) and mean, standard deviation (SD) standard error means are reported, chi-square(X^2) values are reported, and p-values are significant at $p < 0.05$

Table 2 shows the relationship between the socio-demographic factors and the recommended dietary diversity score of the participants. The table also shows the means of the child's age, mother's age, income, and the mean dietary diversity score. The mean age of the children was 11 months. About 65% of the children did not meet the recommended food group intake. A higher proportion of 6-11 months old children (74.0%) had inadequate DD (p<0.001) compared with 12-18 months old children (52.5%). The majority of the children from rural areas (81.8%) could not meet the DD (p<0.001). A greater proportion of children whose mothers were unemployed had inadequate DD (p<0.001).

Table 3: Child morbidity in the past seven days and dietary diversity status of children

**Child morbidity in the past 7 days**	**Inadequate DD (< 9 food groups)**	**Adequate DD (≥ 9 food groups)**	**X^2**	**P-value**
**Running Nose**				
Yes	433 (64.4)	239 (35.6)	0.03	0.871
No	521 (64.9)	282 (35.1)		
**Fever**				
Yes	250 (63.0)	147 (37.0)	0.69	0.425
No	704 (65.3)	374 (34.7)		
**Cough**				
Yes	267 (61.9)	164 (38.1)	1.98	0.168
No	687 (65.8)	357 (34.2)		
**Diarrhoea**				
Yes	190 (66.7)	95 (33.3)	0.61	0.449
No	764 (64.2)	426 (35.8)		
**Vomiting**				
Yes	106 (63.1)	62 (36.9)	0.20	0.668
No	848 (64.9)	459 (35.1)		

Frequency (percentage), chi-square (X^2) P values reported, p-values are significant at $p < 0.05$

Table 3 shows the relationship between child morbidity in the past seven days and the dietary diversity status of infants. From the table, there was no significant difference between child morbidity and dietary diversity score, although most of the children who had no illness for the past seven days recorded a low recommended dietary diversity score. However, children who had diarrhea in the previous week (66.7%) were more likely to have inadequate DD compared with those who had no diarrhea (64.2%). Similarly, children who vomited continuously in the previous week (63.1%) were not likely to consume diverse diet as compared to their counterparts who did not vomit (64.9%).

Table 4: Relationship between maternal nutrition knowledge and dietary diversity status of infants

Mothers' nutrition knowledge	Total for mother's knowledge	Inadequate DD (< 9 food groups)	Adequate DD (9 food groups)	X^2	P-value
Good knowledge		130 (59.9)	87 (40.1)	3.21	0.023
Satisfactory Knowledge		775 (65.3)	412 (34.7)		
Poor knowledge		51 (69.9)	22 (30.1)		
**Mean knowledge score**	**7.0 ± 1.4**				**p<0.001**
When should a woman breastfeed after birth					
Correct		822 (65.4)	435 (34.6)	1.64	0.221
Wrong		134 (60.9)	86 (39.1)		
How long should you exclusively breastfeed without adding any drink or food					
Correct		913 (64.7)	499 (35.3)	0.20	0.630
Wrong		27 (61.4)	17 (38.6)		
How long should you breastfeed before stopping or weaning					
Correct		705 (63.9)	399 (36.1)	1.44	0.231
Wrong		251 (67.3)	122 (32.7)		
When should a woman start to give water after birth					
Wrong		954 (64.7)	521 (35.3)	0.3	0.601
Correct		549 (61.2)	445 (38.8)	0.3	
When should a woman give food after birth?					
Correct		835 (64.8)	454 (35.2)	0.10	0.742
Wrong		117 (63.6)	67 (36.4)		
When should the child start eating family food?					
Correct		551 (61.9)	339 (38.1)	7.66	<0.001
Wrong		386 (69.1)	173 (30.9)		
How much complementary food should one give at 6 months					
Correct		597 (67.9)	282 (32.1)	9.59	<0.001
Wrong		358 (60.1)	238 (39.9)		
How much complementary food should one give a 7 – 8 months					
Correct		260 (62.2)	158 (37.8)	1.62	0.203
Wrong		694 (65.7)	362 (34.3)		
How much complementary food should one give at 9 – 12 months					
Correct		251 (57.8)	183 (42.2)	0.12	0.743
Wrong		297 (56.7)	227 (43.3)		
How many times should you give complementary food at 6 – 8 months					
Correct		683 (64.7)	373 (35.3)	0.00	1
Wrong		271 (64.8)	147 (35.2)		
How many times should you give complementary food at 9 – 12 months					
Correct		104 (55.9)	82 (44.1)	7.11	0.009
Wrong		847 (65.9)	438 (34.1)		
Should the child be fed different kinds of food or the same type					

Correct		924 (64.3)	512 (35.7)	3.81	0.061
Wrong		31 (79.5)	8 (20.5)		

Frequency (percentage), chi-square P values reported, p-values are significant at p < 0.05

Table 4 shows the relationship between nutrition knowledge and the dietary diversity status of the children. The mean nutrition knowledge of the mothers was 7.0 (p<0.001). Children of caregivers who had poor nutrition knowledge (69.9%, P = 0.023) had low dietary diversity scores. Children whose caregivers knew when to provide them with family food (61.9%) had better dietary diversity scores than children whose caregivers did not know when to start giving family food to their children (69.1, p<0.001). Also, caregivers who knew how many times to give complementary food to their children at 9–12 months had their children record better dietary diversity scores (44.1%, 0.00) as compared to their counterparts who did not know (34.1%, p<0.001). However, majority of children of caregivers who knew the quantity of complementary food to give them rather had poor dietary diversity scores (67.9%, p<0.001).

Table 5: Predictors of factors associated with child's dietary diversity

Predictable variable	Adjusted OR (95%CI)	P-value	Unadjusted OR (95%CI)	P-value
**Age**				
12 – 18 months	1		1	
6 – 11 months	2.5 (1.4 – 4.4)	<0.001	2.5 (2.0 - 3.1)	<0.001
**Residence**				
Urban	1		1	
Rural	1.8 (0.1 – 29.1)	0.651	3.2 (2.2 - 4.8)	<0.001
Peri – urban	5.2 (0.2 – 93.2)	0.261	2.8 (2.0 - 3.9)	<0.001
**Employment status**				
Employed	1		1	
Unemployed	1.1 (0.1 – 10.6)	0.925	2.3 (1.7 - 3.2)	<0.001
**Maternal Nutrition knowledge;**				
1. When should a woman give the family food	1.8 (1.0 – 3.2)	0.025	1.3 (1.0 - 1.7)	<0.001
2. how much complementary food should a child eat at 6months	0.9 (0.5 – 1.6)	0.851	1.4 (1.1 - 1.7)	<0.001
3. How many times should you feed a child at 9 months to 12 months	0.9 (0.4 – 2.2)	0.935	1.5 (1.1 - 2.0)	<0.001

OR- Odds ratio, AOR- Adjusted odds ratio, CI- Confidence interval, and p-values are significant at p<0.05, adjusted for gender

Table 5 indicates the predictive factors associated with Child's dietary diversity. Children within 6-11 months (adjusted OR=2.5, p<0.001, 95%CI=1.4 – 4.4) had higher odds of having low dietary diversity compared to the children between 12-18 months. Children who lived in rural (unadjusted OR= 3.2, p<0.001, 95%CI= 2.2-4.8) and peri-urban centers (unadjusted OR= 2.8, p<0.001,

95%CI= 2.0-3.9) had higher odds of having a low dietary diversity as compared to children who lived in urban centers. Children whose mothers were unemployed (unadjusted OR= 2.3, p<0.001, 95%CI= 1.7-3.2) had increased odds of having low dietary diversity as compared to their counterparts whose mothers were employed. Children whose mothers had adequate nutrition knowledge had fewer odds of having lower dietary diversity (Adjusted OR= 0.9, p=0.851, 95%CI = 0.5- 1.6)

4. Discussion

This study identified factors associated with food consumption and dietary diversity among infants in Ghana. Severally implemented policies in Ghana including the National Nutrition Policy has been directed at ensuring the healthy growth and development of infants and young children. However, poor feeding practices are reported among children and young infants in Ghana. From our study, the majority of the children consumed porridge (77.4%) and bread, rice, and noodles (71.6%). A similar study in Ghana reported 81% consumption of bread, noodles, and other grains among children [23]. On the contrary, the intake of milk, vegetables, and meat was very low among the children. Similarly, a study among infants between 6-23 months in rural communities in Ethiopia reported low consumption of animal products including meat and milk [24]. Our study found that more than 6 in 10 (64.7%) children had poor-quality diets. This represents a very low level of dietary diversity among the children. Our findings are in line with a study conducted in Ghana by Bandoh and Kenu, which reported a prevalence of 54% of children not meeting the minimum dietary diversity [25]. A higher prevalence of low dietary diversity was reported among infants between 6-23 months in Tanzania [6]. This indicates that the majority of children in the country are likely to be prone to micronutrient deficiencies. Poor feeding among infants is the cause of infant morbidity and mortality. Therefore, necessary nutrition interventions must be put in place to tackle such issues. A popular approach to curtailing this problem has been the free distribution of nutritionally dense complementary foods or supplements [26].

The findings revealed that some socio-demographic factors were associated with child dietary diversity. Child age was found to be associated with dietary diversity, as 74% of the children who did not meet the minimum dietary diversity were between 6-11 months. According to the study, children in this age group were twice at risk of having a low dietary diversity as compared to their counterparts between 12-18 months. This is consistent with other studies that reported a minimum dietary diversity score among children who were between 6-11 months old in Ghana [27]. The reason is due to the preparedness of older infants to accept food in various forms and their high familiarity with certain foods as compared to younger infants. According to Mura Paroche et al., exposure of an infant to a variety of diets at a young age is efficient in ensuring the liking and consumption of both exposed and new foods [28].

Our study also reported how child residence was a predictor of dietary diversity. About 41% of the children who met the minimum dietary diversity were living in urban centers. Children in rural areas had three times the odds of having a lower dietary diversity than their counterparts in urban centers. Similar findings were seen in a study conducted by [29] among Ghanaian adolescents, where rural dwellers had higher odds of inadequate dietary diversity compared to those in urban centers. A study in Nepal reported a higher prevalence of inadequate dietary diversity among children living in developed areas compared to less developed areas. [30] This can also be seen in

other studies that reported a more diversified food intake among urban residents as compared to rural residents [31, 32]. Urban centers are characterized by food access and high economic status, which may explain the decreased risk of having low dietary diversity as compared to rural areas [33]. Majority of rural areas in Ghana are noted for farming of foodstuffs, fruits and vegetables, however, such cultivation is usually meant for commercial purposes to generate income for the family. Though was not found in the study, certain cultural practices prominent in the rural areas might account for the poor dietary diversity among the children. In certain rural communities, infants are usually not fed with milk with the reason that infants will be spoilt when fed with such non-staple diet [26]. About 37% of pregnant women in a study reported one or more food practices influenced by local beliefs and cultural taboos [34]. Such poor cultural beliefs should not be encouraged since it could hinder the consumption of some micronutrients among infants.

According to our study, maternal employment status is also a predictor of infant dietary diversity. Unemployed women had twice the odds of having a lower dietary diversity score for their children as compared to the other women who were employed. Similar issues were seen in a study conducted by Tampah-Naah, where mothers in Ghana who had challenges accessing complementary food items was due to a lack of income or insufficient financial endowment [35]. In a similar study which reported a low dietary diversity among infants, a higher proportion of mothers could not afford a diverse diet because they had difficulty obtaining a decent income [36]. Interventional policies to engage women in livelihood support, as well as other financial support, are critical to addressing this issue. In 2008, the Livelihood Empowerment Against Poverty was initiated to established in Ghana to improve basic household consumption and nutrition, and increase access to welfare services. Strengthening this policy will provide women access to better livelihood where pregnant women can afford nutrient-dense foods.

According to the study, child morbidity was not a predictor of dietary diversity; however, other studies have reported the contrary. Findings from a study by [37] found that child morbidity was a contributing factor to low dietary diversity. Their study also reported low dietary diversity among children suffering from diarrhea. This is, however, consistent with our study, where children who had diarrhea in the previous week (66.7%) were more likely not to meet the DD compared with those who had no diarrhea (64.2%).

Though certain studies have identified a positive association between maternal education level and child dietary diversity [19,38] our study found no association between maternal educational level and dietary diversity. Similarly, level of education among breastfeeding mothers in India was not associated with breastfeeding practices [39]. However, from our findings, children from mothers who had relatively adequate nutrition knowledge had higher chances of meeting the minimum dietary diversity as compared to the other group. Maternal nutrition knowledge has been established as a predictor of infant feeding practices and nutritional status. A similar observation is seen in a study in Ghana by [40], where households with caregivers who had adequate nutrition knowledge and attitude scores had more diverse diets compared to those with little or no nutrition knowledge. Proper maternal nutrition education interventions are therefore critical to solving such situations. Currently, the Ghana Health Service recommends the intake of a four-star diet for a healthy nutrition. Through the health care systems, pregnant and breastfeeding mothers as part of the antenatal and post-natal services are educated on the consumption of the four-star diet.

Empowering pregnant women with nutrition knowledge will help them make better choices which will eventually ensure a healthy nutrition outcome.

Strengths and limitations of the study

Our study used participants from different socioeconomic setting to determine the factors that were associated with food consumption and dietary diversity among the children as compared to previous studies in Ghana and Sub-Sahara Africa. However, our study was not without limitations. Other factors which can also affect child dietary diversity such as food availability and accessibility and cultural and ethnic factors were not assessed in our study. Moreover, the use of the 24-hour dietary recall is not a true indicator of dietary diversity though it is widely used. However, the 24-hour dietary recall is less subject to recall errors, less cumbersome for the respondent and conforms to the recall time period used in many dietary diversity studies.

Conclusions

The study found that infants had poor-quality diet. The majority of the infants also consumed fewer food groups from the 18-food group list. Significant factors associated with poor dietary diversity were the child's age, maternal employment status, residence, and maternal nutrition knowledge. Our study has shown that the factors associated with poor dietary diversity among the infants were multifactorial ranging from age-specific, occupation, nutritional knowledge of mother and residence of infants. Based on the findings in this study, we suggest that proper maternal nutrition education and feeding practices targeting age-specific needs and community livelihood support systems are necessary to improve dietary diversity of infants.

Data Availability

Data cannot be shared publicly due to the fact that it contains sensitive identifying information. However, data are available from the Committee on Human Research, Publication, and Ethics (CHRPE), the ethics board of the School of Medicine and Dentistry of the Kwame Nkrumah University of Science and Technology (KNUST), and Komfo Anokye Teaching Hospital (KATH), Kumasi, Ghana (email: chrpe.knust.kath@gmail.com) for researchers who meet the criteria for access to confidential data.

Funding

The authors received no specific funding for this study.

Acknowledgment

We thank the directors of health and health workers in the health centers where the study took place and most of all the mothers and infants who participated in the study.

Authors contribution

Conceptualization: Reginald Adjetey Annan, Godwin Opoku Agyemang,

Data curation: Godwin Opoku Agyemang, Reginald Adjetey Annan, Samuel Selorm Attu, Odeafo Asamoah Boakye

Formal analysis: Godwin Opoku Agyemang, Reginald Adjetey Annan, Odeafo Asamoah-Boakye

Fund acquisition: Reginald Adjetey Annan, Godwin Opoku Agyemang

Investigation: Godwin Opoku Agyemang, Reginald Adjetey Annan

Project administration: Reginald Adjetey Annan, Godwin Opoku Agyemang, Samuel Selorm Attu

Resources: Reginald Adjetey Annan, Godwin Opoku Agyemang

Software: Reginald Adjetey Annan, Godwin Opoku Agyemang, Odeafo Asamoah-Boakye

Supervision: Reginald Adjetey Annan, Satoru Okonogi, Takeshi Sakurai

Writing- Review and editing: Reginald Adjetey Annan, Godwin Opoku Agyemang, Odeafo Asamoah-Boakye, Satoru Okonogi, Takeshi Sakurai, Samuel Selorm Attu

REFERENCES

1. Gizaw G, Tesfaye G. Minimum acceptable diet and factor associated with it among infant and young children age 6-23 months in north Shoa, Oromia region, Ethiopia. Int J Homeopathy Nat Med. 2019;5(1):1. doi:10.11648/j.ijhnm.20190501.11

2. United Nations Children's Fund (UNICEF), World Health Organization (WHO) & International Bank for Reconstruction and Development/The World Bank (2020) Levels and Trends in Child Malnutrition: Key Findings of the 2020 Edition of the Joint Child Malnutrition Estimates.
3. Ng CS, Dibley MJ, Agho KE. Complementary feeding indicators and determinants of poor feeding practices in Indonesia: a secondary analysis of 2007 Demographic and Health Survey data. Public health nutrition. 2012 May;15(5):827-39. https://doi.org/10.1017/s1368980011002485
4. Eshete T, Kumera G, Bazezew Y, Mihretie A, Marie T. Determinants of inadequate minimum dietary diversity among children aged 6–23 months in Ethiopia: secondary data analysis from Ethiopian Demographic and Health Survey 2016. Agriculture & Food Security. 2018 Dec;7(1): https://doi.org/10.1186/s40066-018-0219-8

5. WHO. Infant and young child feeding. 2018. Available through https://www.who.int/news-room/fact-sheets/detail/infant-and-young-child-feeding. Retrieved on 21 February 2019.
6. Khamis AG, Mwanri AW, Ntwenya JE, Kreppel K. The influence of dietary diversity on the nutritional status of children between 6 and 23 months of age in Tanzania. BMC pediatrics. 2019 Dec;19(1):1-9. https://doi.org/10.1186/s12887-019-1897-5

7. Frempong RB, Annim, S K: Dietary diversity and child malnutrition in Ghana. *Heliyon*, (2017). *3*(5), e00298. https://doi.org/10.1016/j.heliyon.2017.e00298

8. Nti CA: Dietary diversity is associated with the nutrient intake and nutritional status of children in Ghana. *Asian Journal of Medical Sciences*, (2011).*2*(2), 105-109.

9. Onyango AW. Dietary diversity, child nutrition and health in contemporary African communities. Comparative biochemistry and physiology part A: Molecular & Integrative Physiology. 2003 Sep 1;136(1):61-9. https://doi.org/10.1016/s1095-6433(03)00071-0

10. World Health Organization. (2008). *Indicators for assessing infant and young child feeding practices part 3: country profiles*. World Health Organization.

11. World Health Organization (WHO) (2008) Indicators for Assessing Infant and Young Child Feeding Practices: Part 1.

12. Jalata DD, & Asefa B: Trends and Determinants of Dietary Diversity in Children Aged 6–59 Months in Ethiopia (2022) Analysis of 2005–2016 Demographic and Health Survey. *Current Developments in Nutrition*, *6*(10), nzac135. https://doi.org/10.1093/cdn/nzac135

13. Wiafe MA, Apprey C, Annan RA:.Dietary diversity and nutritional status of adolescents in rural Ghana. *Nutrition and Metabolic Insights*, (2023) *16*, 11786388231158487. https://doi.org/10.1177/11786388231158487

14. Saaka M, Larbi A, Mutaru S, Hoeschle-Zeledon, I: Magnitude and factors associated with appropriate complementary feeding among children 6–23 months in northern Ghana. *BMC Nutrition*, (2016)*2*, 1-8. https://doi.org/10.1186/s40795-015-0037-3

15. Annan RA, Apprey C, Asamoah- Boakye O, Okonogi S, Yamauchi T, Sakurai T: The relationship between dietary micronutrient intake and cognition test performance among school- aged children in government- owned primary schools in Kumasi metropolis, Ghana. *Food Science & Nutrition*, (2019)*7*(9), 3042-3051. https://doi.org/10.1002/fsn3.1162

16. Annan RA, Apprey C, Odeafo AB, Benedicta TD, Sakurai T, Okonogi S. Nutrient intakes and cognitive competence in the context of abstract reasoning of school-age children in the Tamale Metropolis of Ghana. Nutrition & Food Science. 2023 Jan 2;53(1):124-37. https://doi.org/10.1108/NFS-11-2021-0343

17. Sirait AR, Achadi EL. Factors Associated with Minimum Dietary Diversity among Breas tfed Children Aged 6-23 Months in Indonesia (Analysis of Indonesia DHS 2017). Indonesian Journal of Public Health Nutrition. 2020 Oct 30;1(1). http://dx.doi.org/10.7454/ijphn.v1i1.4381

18. Erismann S, Knoblauch AM, Diagbouga S, Odermatt P, Gerold J, Shrestha A, et al. Prevalence and risk factors of undernutrition among schoolchildren in the Plateau Central and Centre-Ouest regions of Burkina Faso. Infect Dis Poverty. 2017; 6(1): 17. https://doi.org/10.1186/s40249-016-0230-x

19. Agrawal S, Kim R, Gausman J, Sharma S, Sankar R, Joe W, Subramanian SV. Socio-economic patterning of food consumption and dietary diversity among Indian children: evidence from NFHS-4. European Journal of Clinical Nutrition. 2019 Oct;73(10):1361-72. https://doi.org/10.1038/s41430-019-0406-0

20. Shariff ZM, Lin KG, Sariman S, Lee HS, Siew CY, Yusof BN, Mun CY, Mohamad M. The relationship between household income and dietary intakes of 1-10 year old urban Malaysian. Nutrition research and practice. 2015 Jun 1;9(3):278-87. https://doi.org/10.4162/nrp.2015.9.3.278

21. Saaka M, Awini S, Nang E. Prevalence and predictors of appropriate complementary feeding practice among mothers with children 6–23 months in Northern Ghana. World Nutrition. 2022 Jun 30;13(2):14-23. https://doi.org/10.26596/wn.202213214-23

22. Food Agriculture Organization (FAO); Guidelines for measuring household and individual dietary diversity; 2010

23. Anane, I., Nie, F., & Huang, J. (2021). Socioeconomic and geographic pattern of food consumption and dietary diversity among children aged 6–23 months old in Ghana. *Nutrients*, *13*(2), 603. https://doi.org/10.3390/nu13020603

24. Haileselassie M, Redae G, Berhe G, Henry CJ, Nickerson MT, Tyler B, Mulugeta A. Why are animal source foods rarely consumed by 6-23 months old children in rural communities of Northern Ethiopia? A qualitative study. PloS one. 2020 Jan 8;15(1):e0225707. https://doi.org/10.1371/journal.pone.0225707

25. Bandoh DA, Kenu E. Dietary diversity and nutritional adequacy of under-fives in a fishing community in the central region of Ghana. BMC Nutrition. 2017 Dec;3:1-6. https://doi.org/10.1186/s40795-016-0120-4

26. Okonogi S, Annan R, Sakurai T. Improving Infant Nutrition through the Market: Experimental Evidence in Ghana. Available at SSRN 3771891. 2021 Jul 8. https://dx.doi.org/10.2139/ssrn.3771891

27. Issaka AI, Agho KE, Burns P, Page A, Dibley MJ. Determinants of inadequate complementary feeding practices among children aged 6–23 months in Ghana. Public health nutrition. 2015 Mar;18(4):669-78. https://doi.org/10.1017/s1368980014000834
28. Mura Paroche M, Caton SJ, Vereijken CM, Weenen H, Houston-Price C. How infants and young children learn about food: A systematic review. Frontiers in Psychology. 2017 Jul 25;8:1046. https://doi.org/10.3389/fpsyg.2017.01046
29. Gyimah LA, Annan RA, Apprey C, Edusei A, Aduku LN, Asamoah-Boakye O, Azanu W, Lutterodt H. Dietary diversity and its correlates among pregnant adolescent girls in Ghana. PLoS One. 2021 Mar 8;16(3):e0247979. https://doi.org/10.1371/journal.pone.0247979
30. Baek Y, Chitekwe S. Sociodemographic factors associated with inadequate food group consumption and dietary diversity among infants and young children in Nepal. PloS one. 2019 Mar 11;14(3):e0213610. https://doi.org/10.1371/journal.pone.0213610
31. Paramashanti BA, Huda TM, Alam A, Dibley MJ. Trends and determinants of minimum dietary diversity among children aged 6–23 months: a pooled analysis of Indonesia demographic and health surveys from 2007 to 2017. Public Health Nutrition. 2022 Jul;25(7):1956-67. https://doi.org/10.1017/s1368980021004559
32. Amugsi DA, Mittelmark MB, Lartey A. Dietary diversity is a predictor of acute malnutrition in rural but not in urban settings: evidence from Ghana. Child Care Practices, Resources for Care, and Nutritional Outcomes in Ghana: Findings from Demographic and Health Surveys. 2014 Jun 5.
33. Wang H, Liu C, Fan H, Tian X. Rising food accessibility contributed to the increasing dietary diversity in rural and urban China. Asia Pacific journal of clinical nutrition. 2017 Jan;26(4):738-47. https://doi.org/10.6133/apjcn.052016.03
34. Chakona G, Shackleton C. Food taboos and cultural beliefs influence food choice and dietary preferences among pregnant women in the Eastern Cape, South Africa. Nutrients. 2019 Nov 5;11(11):2668. https://doi.org/10.3390/nu11112668
35. Tampah-Naah AM, Kumi-Kyereme A, Amo-Adjei J. Maternal challenges of exclusive breastfeeding and complementary feeding in Ghana. PloS one. 2019 May 2;14(5):e0215285. https://doi.org/10.1371/journal.pone.0215285

36. Chakona G. Social circumstances and cultural beliefs influence maternal nutrition, breastfeeding and child feeding practices in South Africa. Nutrition Journal. 2020 Dec;19(1):1-5. https://doi.org/10.1186/s12937-020-00566-4
37. Rah JH, Akhter N, Semba RD, De Pee S, Bloem MW, Campbell AA, Moench-Pfanner R, Sun K, Badham J, Kraemer K. Low dietary diversity is a predictor of child stunting in rural Bangladesh. European journal of clinical nutrition. 2010 Dec;64(12):1393-8. https://doi.org/10.1038/ejcn.2010.171
38. Sekartaji R, Suza DE, Fauziningtyas R, Almutairi WM, Susanti IA, Astutik E, Efendi F. Dietary diversity and associated factors among children aged 6–23 months in Indonesia. Journal of Pediatric Nursing. 2021 Jan 1;56:30-4. https://doi.org/10.1016/j.pedn.2020.10.006
39. Ghosh P, Rohatgi P, Bose K. Determinants of time-trends in exclusivity and continuation of breastfeeding in India: An investigation from the National Family Health Survey. Social Science & Medicine. 2022 Jan 1;292:114604. https://doi.org/10.1016/j.socscimed.2021.114604
40. Christian AK, Marquis GS, Colecraft EK, Lartey A, Sakyi-Dawson O, Ahunu BK, Butler LM. Caregivers' nutrition knowledge and attitudes are associated with household food diversity and children's animal source food intake across different agro-ecological zones in Ghana. British Journal of Nutrition. 2016 Jan;115(2):351-60. https://doi.org/10.1017/s0007114515004468

Supplementary Table 1: Food group consumption by age groups and dietary diversity of child

Age group (Months)	Food consumption		Dietary diversity score		X^2	P-value
			**Not met DDS (< 9 food groups)**	**met DDS (≥ 9 food groups)**		
Bread, rice, noodles						
6 – 11	Yes		324 (61.0)	302 (39.0)	212.94	<0.001
	No		298 (96.1)	12 (3.9)		
12 – 18	Yes		240 (45.5)	287 (54.5)		
	No		15 (13.8)	94 (86.2)		
Cassava, Plantain, cocoyam						
6 – 11	Yes		75 (58.1)	54 (41.9)	119.11	<0.001
	No		568 (79.8)	144 (20.2)		
12 – 18	Yes		75 (37.7)	128 (62.3)		
	No		259 (59.3)	178 (40.7)		
Porridge						
6 – 11	Yes		448 (69.0)	201 (31.0)	55.26	<0.001
	No		174 (90.6)	18 (9.4)		
12 – 18	Yes		234 (47.5)	259 (52.5)		
	No		100 (69.9)	43 (30.1)		
Any oils (margarine, butter, etc						
6 – 11	Yes		137 (50.2)	136 (49.8)	275.46	<0.001
	No		485 (85.4)	83 (14.6)		
12 – 18	Yes		95 (30.1)	221 (69.9)		
	No		239 (74.7)	81 (25.3)		
Sugary foods (sweet, chocolate)						
6 – 11	Yes		158 (54.5)	132 (45.5)	210.84	<0.001
	No		464 (84.2)	87 (15.8)		
12 – 18	Yes		108 (33.3)	216 (66.7)		
	No		226 (72.4)	86 (27.6)		
Infant formula						
6 – 11	Yes		229 (63.1)	134 (36.9)	43.40	<0.001
	No		393 (82.2)	85 (17.8)		
12 – 18	Yes		66 (36.1)	117 (63.9)		
	No		268 (59.2)	185 (40.8)		
Tinned powdered or fresh animal milk						
6 – 11	Yes		152 (54.1)	129 (45.9)	213.44	<0.001
	No		470 (83.9)	90 (16.1)		
12 – 18	Yes		334 (52.5)	302 (47.5)		
	No		241 (71.7)	95 (28.3)		
Eggs						
6 – 11	Yes		194 (54.3)	163 (45.7)	270.50	<0.001
	No		428 (88.4)	56 (11.6)		

12 – 18	Yes		170 (38.8)	268 (61.2)		
	No		164 (82.8)	34 (17.2)		
Fresh or dried fish						
6 – 11	Yes		237 (55.5)	190 (44.5)	253.91	<0.001
	No		358 (93.0)	29 (7.0)		
12 – 18	Yes		209 (43.4)	273 (56.6)		
	No		125 (81.2)	29 (18.8)		

Frequency (percentage), Fisher's exact test P values reported, p-values are significant at p < 0.05

Supplementary Table 2: Food group consumption by age groups and dietary diversity of child

Age group (Months)	Food Group	Dietary diversity score		X^2	P-value
		**Not met DDS (< 9 food groups)**	**met DDS (≥ 9 food groups)**		
Organ meat					
6 – 11	Yes	27 (32.5)	56 (67.5)	201.30	<0.001
	No	595 (78.5)	163 (21.5)		
12 – 18	Yes	17 (13.6)	108 (86.4)		
	No	317 (62.0)	194 (38.0)		
Any meat					
6 – 11	Yes	51 (35.7)	92 (64.3)	302.42	<0.001
	No	571 (81.8)	127 (18.2)		
12 – 18	Yes	58 (24.0)	184 (76.0)		
	No	276 (70.1)	118 (29.9)		
Foods made from beans, lentils, etc.					
6 – 11	Yes	76 (36.5)	132 (63.5)	140.82	<0.001
	No	546 (86.3)	87 (13.7)		
12 – 18	Yes	44 (20.1)	175 (79.9)		
	No	290 (69.5)	127 (30.5)		
Yogurt or cheese					
6 – 11	Yes	84 (43.3)	110 (56.7)	309.94	<0.001
	No	538 (83.2)	109 (16.8)		
12 – 18	Yes	48 (20.3)	189 (79.7)		
	No	286 (71.7)	113 (28.3)		
Fruits					
6 – 11	Yes	190 (53.1)	168 (46.9)	303.33	<0.001
	No	432 (89.4)	51 (10.6)		
12 – 18	Yes	131 (34.0)	254 (66.0)		
	No	203 (80.9)	48 (19.1)		

Carrots or sweet potato					
6 – 11	Yes	16 (21.3)	59 (78.7)	173.71	<0.001
	No	606 (79.1)	160 (20.9)		
12 – 18	Yes	15 (16.5)	76 (83.5)		
	No	319 (58.5)	226 (41.5)		
Dark green leafy vegetables					
6 – 11	Yes	206 (53.8)	177 (46.2)	268.36	<0.001
	No	416 (90.8)	42 (9.2)		
12 – 18	Yes	131 (35.4)	239 (64.6)		
	No	203 (76.3)	63 (23.7)		
Ripe mangoes or pawpaw					
6 – 11	Yes	17 (20.7)	65 (79.3)	211.84	<0.001
	No	605 (79.7)	154 (20.3)		
12 – 18	Yes	21 (17.8)	97 (82.2)		
	No	313 (60.4)	205 (39.6)		
Any other fruits and vegetables					
6 – 11	Yes	116 (44.1)	147 (55.9)	81.95	<0.001
	No	506 (87.5)	72 (12.5)		
12 – 18	Yes	80 (30.9)	179 (69.1)		
	No	254 (67.4)	123 (32.6)		

Frequency (percentage), Fisher's exact test P values reported, p-values are significant at $p < 0.05$

Regression Analysis showing the predictors of dietary diversity.

Variables in the Equation

		B	S.E.	Wald	df	Sig.	Exp(B)	95% C.I.for EXP(B)	
								Lower	Upper
Step 1[a]	educational_level	.293	.177	2.750	1	.097	1.340	.948	1.895
	INCOMESTATUS	-.215	.225	.915	1	.339	.807	.519	1.253
	Childs_age(1)	.928	.288	10.375	1	.001	2.529	1.438	4.448
	resident_REGRESSION			1.311	2	.519			
	resident_REGRESSION (1)	.628	1.399	.201	1	.654	1.873	.121	29.070
	resident_REGRESSION (2)	1.652	1.471	1.261	1	.261	5.217	.292	93.254
	employment_REGRESSI ON(1)	.105	1.155	.008	1	.928	1.110	.115	10.676
	WHEN_GIVE_FAMILY_FO OD_GRADE(1)	.634	.277	5.220	1	.022	1.885	1.094	3.246
	HOW_MUCH_COMPLEM ENTARY_FOOD_SHOUL D_A_CHILD_EAT_AT_6M ONTHS(1)	-.049	.268	.033	1	.855	.952	.563	1.610
	HOWMANYTIMESFEEDA CHILDFROM9TO12MONT HS(1)	-.038	.439	.007	1	.931	.963	.407	2.278
	Constant	-.709	.634	1.248	1	.264	.492		

a. Variable(s) entered on step 1: Childs_age, resident_REGRESSION, employment_REGRESSION, WHEN_GIVE_FAMILY_FOOD_GRADE, HOW_MUCH_COMPLEMENTARY_FOOD_SHOULD_A_CHILD_EAT_AT_6MONTHS, HOWMANYTIMESFEEDACHILDFROM9TO12MONTHS.

Variables in the Equation

		B	S.E.	Wald	df	Sig.	Exp(B)	95% C.I.for EXP(B)	
								Lower	Upper
Step 1[a]	employment_REGRESSION (1)	.865	.156	30.765	1	.000	2.375	1.750	3.225
	Constant	.460	.074	38.828	1	.000	1.583		

a. Variable(s) entered on step 1: employment_REGRESSION.

Variables in the Equation

		B	S.E.	Wald	df	Sig.	Exp(B)	95% C.I.for EXP(B)	
								Lower	Upper
Step 1[a]	resident_REGRESSION			67.033	3	.000			
	resident_REGRESSION(1)	1.188	.200	35.324	1	.000	3.279	2.216	4.851
	resident_REGRESSION(2)	1.055	.168	39.648	1	.000	2.873	2.068	3.989
	resident_REGRESSION(3)	20.886	40192.970	.000	1	1.000	1177104800.63 4	.000	.
	Constant	.317	.063	25.219	1	.000	1.372		

a. Variable(s) entered on step 1: resident_REGRESSION.

Variables in the Equation

		B	S.E.	Wald	df	Sig.	Exp(B)	95% C.I.for EXP(B)	
								Lower	Upper
Step 1[a]	Childs_age(1)	.943	.112	71.282	1	.000	2.568	2.063	3.197
	Constant	.101	.079	1.609	1	.205	1.106		

a. Variable(s) entered on step 1: Childs_age.

One-Sample Statistics

	N	Mean	Std. Deviation	Std. Error Mean
age of target child	1503	11.03	3.306	.085
maternal age	1368	30.16	11.977	.324
TOTALKNOWLEDGESCORE	1503	7.00	1.497	.039
total_DDscore	1477	7.04	3.830	.100

Mean_Dietary diversity score

		Frequency	Percent	Valid Percent	Cumulative Percent
Valid	Above 9 DDS	521	34.7	35.3	35.3
	Below 9 DDS	956	63.6	64.7	100.0
	Total	1477	98.3	100.0	
Missing	System	26	1.7		
Total		1503	100.0		

Department of Biochemistry,

College of Science, KNUST,

Kumasi, Ghana.

Pradip Chouhan, PhD

Academic Editor

PLOS ONE

Dear Academic editor,

POINT-BY-POINT REVISIONS IN RESPONSE TO REVIEWERS' COMMENTS

In line with the reviewer's comments, the following revisions have been made to the manuscript ID: PONE-D-23-17357 entitled 'Factors associated with food consumption and dietary diversity among infants aged 6-18 months in Ashanti Region Ghana' by Godwin Opoku Agyemang, Samuel Selorm Attu, Reginald Annan, Satoru Okonogi, Takeshi Sakura and Odeafo Asamoah-Boakye.

Below is our submission;

Response to Reviewer 2

1. In the Materials and Methods section of the manuscript, keep only "Dependent variable:" and "Independent variable:" labeled as point. and remove the word "Data collection." Remove "socio-demographic," "complementary feeding knowledge," "complementary feeding practices," "child morbidity," and "dietary diversity" after "Dependent variable:" and "Independent variable:

   Response: Please the Materials and Methods section of the manuscript have been revised accordingly. The dependent and independent variable have clearly been stated. The above said words have been removed accordingly. Please kindly refer to page 4 for corrections made.

2. In the "Strengths and Limitations of the study" section, remove the phrases "a very large sample size and" and "with a relatively smaller sample size" from the first sentence.

Response: Please the above phrases have been removed from the "Strengths and limitations" section on page 13.

3. Check and very ole literatures should be replaced by the updated ones.

   Response: References in the study have been revised. Very old references are replaced with new ones with majority spanning from the past 6 years and few in the past 10 years.

4. Include DOI or link to each reference.

   Response: Please a DOI has been added to majority of the references except those that had no DOI or link to them.
