# Supplementary material for: Factors associated with food consumption and dietary diversity among infants aged 6–18 months in Ashanti Region, Ghana
Source: PLoS One. 2023 Nov 30;18(11):e0294864. doi: 10.1371/journal.pone.0294864 (PMC10688859; doi:10.1371/journal.pone.0294864)
Supplement: Attachment — Submitted filename: Response to reviewers.docx [file pone.0294864.s003.docx]

Department of Biochemistry,

College of Science, KNUST,

Kumasi, Ghana.

Pradip Chouhan, *PhD*

Academic Editor

PLOS ONE

Dear Academic editor,

**POINT-BY-POINT REVISIONS IN RESPONSE TO REVIEWERS’ COMMENTS**

In line with the reviewer’s comments, the following revisions have been made to the manuscript ID: PONE-D-23-17357 entitled ‘Factors associated with food consumption and dietary diversity among infants aged 6-18 months in Ashanti Region Ghana’ by Godwin Opoku Agyemang, Samuel Selorm Attu, Reginald Annan, Satoru Okonogi, Takeshi Sakura and Odeafo Asamoah-Boakye.

Below is our submission;

**Journal Requirements**

1. Our revised manuscripts meet PLOS ONE’s style requirements, including those for file namings, and other formatting styles
2. Financial disclosure: **The authors received no specific funding for this work**. The study was assigned to master’s students for their project thesis. Therefore, funding was sourced solely by the students on the project.
3. Data for this study is available with the Committee on Human Research Publication and Ethics, KNUS, Komfo Anokye Teaching Hospital (KATH), and also the principal investigator which will be made available upon request

**REVIEWER #1**

**Comment 1:**

Abstract:

Well Written. Need to rewrite the background part of the abstract. Try to address objective of the study in abstract part.

**Response 1:** The background part of the abstract has been restructured to reflect the objective of the study. The concept of food consumption and dietary diversity which is the main focus of the study have been incorporated in the background. It is currently captured as “*Studies have reported a poor diet quality among children in Ghana and other developing countries. Inadequate dietary diversity among children may lead to deficiencies in micronutrient intake necessary for growth and other cognitive functions. understanding factors associated with infants’ diverse diets is a key step to promoting adequate infant and young child nutrition. This study sought to determine the factors associated with food consumption and dietary diversity among infants*”

**Comment 2**

Introduction: Need to improve.

The research gap is unclear. Overall, the introduction is unclear as to what the authors want to say. It is better to rewrite based on i) what the issue is, ii) why it is important, iii) what the literature says, iv) what the research gap and question in this study are, and v) how this study could contribute to the literature.

**Response 2**

Please the entire introduction has been modified accordingly. The first paragraph of the introduction talks about the problem of poor dietary diversity among infants globally, sub-Sahara Africa and Ghana. The paragraph also gives some trends of poor dietary diversity in Africa and Ghana. The second paragraph explains dietary diversity and its relevance to infants’ health. The second paragraph highlights how poor dietary diversity among infants can present a lot of deficiencies in micronutrients intake which can hinder growth. Current literature reporting the factors associated with dietary diversity has been highlighted in the third paragraph. Factors including socioeconomic, nutrition knowledge, micronutrient intake and household food security have been reported by other studies. The third paragraph also compares the methods employed in previous studies and the research gap which was targeted in our study. Data on this subject matter is insufficient in Ashanti Region which is noted for its extreme diversity in ethnicity, socioeconomic status, and food variations. And this point was stated in the introduction. Conducting such a large population research in the second most populous city in Ghana will serve as a basis for policy implementation and that was mentioned in the introduction. Please see page 2 and 3 for more details.

**Comment 3** The concept, methods and results are cohesive and scientifically sound. If I would nit-pick, there are some issues with grammar. However, the manuscript can be understood regardless.

**Response 3**. Grammatical errors have been corrected in the entire manuscript. The work has been proof-read and grammatical errors including punctuations and wrong statements have been corrected

**Comment 4**

Yet, to be of interest for readers outside Ghana, too, the paper should also refer to some studies in the international literature (studies done on the children of others countries) on child feeding practice, to highlight the gaps and unanswered questions, the methodologies used etc. Authors may cite following literature on breastfeeding practice in India: *Ghosh P, Rohatgi P, Bose K. Determinants of time-trends in exclusivity and continuation of breastfeeding in India: An investigation from the National Family Health Survey. Social Science & Medicine. 2022 Jan 1;292:114604*.

**Response 4:** Please other internationally related studies have been cited in this work. The prevalence of dietary diversity has been compared with other studies conducted in various parts of Africa and Asia. This has been done to all the aspects of the study including the associated factors of dietary diversity. The authors also found the above suggested citation relevant and has been cited in our study [Please see page 14, reference:39]. The various methods which were employed in previous studies have been varied with our present study and explanation has been given. Please see page 14.

**Comment 5**

The Discussion section tells a coherent story of what was discovered and the implications for Ghana. I suggest the authors to shed light on those results that may be generalizable (outside the Ghana context). This section should also reflect the contribution of the study to the international literature in the study area.
b) In this section authors have already summarized the process, the results, and the overall purpose of this study.
c) One paragraph/part should answer questions about the limitations and potential flaws or shortcomings of this study. One paragraph should focus on the successes of the study.
d) Authors can also compare the results of different methods and assess which was more fruitful and why. Discuss the implications of this research and compare it to the results of other studies. Authors can cite related studies to show how your study compares (in addition to the introduction).

**Response 5:** Please the discussion has been reviewed accordingly based on the above suggested points. The outcome of the study was well discussed and compared with similar studies outside Ghana and the complications of the findings were elaborated. The various methods employed in the previous studies were considered and discussed in comparison to our study. Limitations and potential flaws of the study has been included in the discussion. The study reported the use of the 24-hour dietary recall as one of the flaws of the study however, due to its less cumbersome nature, less subject to recall errors and its conformation to the recall time period used in many diversity studies, it was used. Also, other factors known to affect dietary diversity such as food availability, cultural and ethnic factors among others were not reported in our study, however, these factors were discussed under the discussion section. Please see page 15 for details.

**Comments 6**

Conclusion: The conclusion offers you a chance to briefly present how your work advances the field from the present state of knowledge. Make a proper justification for your work here and indicate extensions and wider implications of the study.

**Response 6:** The conclusions have been revised accordingly. From our study, the infants had poor-quality diet as majority of them consumed fewer food groups than the recommended. Factors associated with the poor dietary diversity included child’s age, maternal employment status, residence and nutrition knowledge. Recommendations were made under this section. Please see revision made under the conclusion at page 14

**REVIEWER #2**

**Comment 1:**

The rationale behind selecting the Ashanti Region in Ghana as the study area needs to be explained and justified by the author.

**Response 1:**

Ashanti Region is the second most populous city in Ghana noted for its ethnic diversity. Also noted for its farming activities, the region has viable lands which support the cultivation of several crops, fruits and vegetables. It is also noted for economic and other trading activities. Therefore, it is believed that with the availability and access to the different food groups in the region, genuine factors associated with dietary diversity will be determined. Also, the presence of different ethnic groups in the region could be a general representation of the entire country. Some studies about child’s feeding behaviors have been done in the Ashanti region. Please see the justification in the Methods section [Page 4].

**Comment 2:**

The research paper lacks novelty, and it is necessary to incorporate this in the end of the introduction section of the manuscript.

**Response 2**: geographical settings and cultural barriers are known to be associated with dietary diversity. To our best of knowledge, fewer studies have focused on the geographical factors including rural, urban and peri urban among infants’ dietary diversity. This study used a large population (more than 1000 participants) from different geographical setting, ethnicities, communities and socioeconomic background to report factors associated with food consumption and dietary diversity among infants 6-18 months. The large population of this study has influenced the statistical strength of the data collected. This point has been stated in the last paragraph of the introduction in page 3. Also, the setting for the study exhibits a wide range of regional diversity and differences in terms of socio-economy, quality of life, human development and demography. Reason for the selection of the site for the study is well explained under the method section on page 4.

**Comment 3:**

Check spelling/writing whole manuscript. Several errors are available in the manuscript (IN ASHANTI REGION GHANA…. (77.4%)… (35%)

**Response 3:** Please grammatical errors in the manuscripts have been corrected.

**Comment 4:**

Please rewrite the whole “materials and methods” section. I am unable to understand how many independent and dependent variables there are. Do not make it complicated. Try to simplify.

**Response 4**: Please the material and methods section has been revised accordingly. The study design and sampling have been clearly written. Both the dependent and independent variables have been clearly defined and simplified. The independent variables in the study were socio-demographic, maternal knowledge and practices, and child morbidity. The dependent variable in the study was dietary diversity. How all these variables were assessed have been simplified under each section on page 4 and page 5.

**Comment 5:**

The author refers to the prevalence of child dietary diversity, but unfortunately, I couldn't locate the specific table for this.

**Response 5**

Please the prevalence of dietary diversity (DD) is highlighted in Table 2: Relationship between the sociodemographic factors and the recommended dietary diversity score of inf ants. 64.7% of the infants did not meet the minimum dietary diversity. This can be found on page 9. However, another figure has been included which clearly represents the dietary diversity among the children.

**Comment 6**:

Figure 1 is not clearly visible.

**Response 6:** Please the visibility of Figure 1 has been enhanced.

**Comment 7**:

How did you select the sample for your study? Could you please provide details about the procedure used for sample selection?

**Response 7**: Sampling method and procedure has been explained in the Methods section, page 4 under the study population and sampling. *“A total of 28 CWCs were selected from a total of 40. All mothers whose infants were aged 6–18 months and were attending the selected CWC were recruited during the four weeks of the recruitment period. The mothers were conveniently sampled during their routine visits to the community weighing centers (CWC) in their communities. Mothers who had twin infants and those whose children were outside the age bracket of the study were excluded. The date of birth (day/month/year) of infants, which was recorded in the child’s weight records books, was used to determine the infants ages. A list of mother-infant (6–18 months) pairs from the community weighing centers (CWC) was generated, and every child within the selected age range was eligible for inclusion”*

**Comment 8:**

Please include detailed analytical techniques (formulas, descriptions etc.) for "Dominance analysis of relative contribution" and "Binary logistic regression" in the appendix for the reader.

**Response 8:** Some analysis which were conducted in the study including the binary logistic regression has been included in the appendix.

**Comment 9:**

I couldn't find any information regarding existing policies or policy enhancement strategies in the discussion section of this study related to food consumption and dietary diversity among infants aged 6-18 months in Ghana.

**Response 9:** please the discussion section has been revised accordingly. Relevant policies and interventions related to food consumption and dietary diversity in Ghana has been included in the discussion section. In 2008, the Livelihood Empowerment Against Poverty (LEAP) was initiated in Ghana to improve the basic household consumption and nutrition, and increases access to welfare services. Having reported a positive association between maternal employment status and infant dietary diversity, the strengthening the LEAP policy among household will provide a source of income for women to afford a diverse diet for their wards [paragraph 3 of discussion]. Though no association was found between maternal education and dietary diversity, maternal nutrition knowledge was found to be associated with child dietary diversity. From our study, the policy by the Ghana Health Service which recommends the intake of a four-star diet for a healthy nutrition should be well-thought in the antenatal and post-natal services. This will help them make better choices [last paragraph of discussion]. Please see page 14 for details

**Comment 10**:

Other necessary factors that impact food consumption and dietary diversity among infants aged 6-18 months should be considered. These factors include maternal and family support, parental feeding practices (such as pressure to eat), food availability and accessibility, and cultural and ethnic factors. Please add.

**Response 10:** Please the discussion has be revised to include the above factors which can impact food consumption and dietary diversity among the infants. In our study, children from rural areas had higher odds of not meeting the dietary diversity. Factors including poor cultural beliefs was discussed to be the reason for this. Certain cultures in Ghana predominantly in rural areas of Ghana prohibits children from being fed with milk and this can hinder micronutrient intake [See last part of paragraph 3 under the discussion]. Though not reported in the results these factors were reported in the study. Other factors including food availability and parental feeding parental feeding practices are reported under the discussion section.

**Comment 11:**

What are the strength and limitations of the study? Please mention.

**Response 11:** The strength and Limitation of the study has been stated on page 14 as *“Our study used a very large sample size and participants from different socioeconomic setting to determine the factors that were associated with food consumption and dietary diversity among the children as compared to previous studies in Ghana and Sub-Sahara Africa with relatively smaller sample size. However, our study was not without limitations. Other factors which can also affect child dietary diversity such as food availability and accessibility and cultural and ethnic factors were not assessed in our study. Moreover, the use of the 24-hour dietary recall is not a true indicator of dietary diversity though it is widely used. However, the 24-hour dietary recall is less subject to recall errors, less cumbersome for the respondent and conforms to the recall time period used in many dietary diversity studies”.*

**Comment 12:**

In the discussion section, it is unnecessary to restate the result of the study. “(unadjusted OR=2.3, p. Please remove.

**Response 12**: Thank you for this correction. The suggestion has been corrected under the discussion section.

**Comment 13**:

What interventions and strategies can be implemented to enhance food consumption and dietary diversity among infants aged 6-18 months in Ghana? Please recommend this in the conclusion section of the manuscript.

**Response 13:** Please interventions and strategies to improve food consumption and dietary diversity among infants have been included in the conclusion section of the manuscript. “*Proper maternal education and the provision of community livelihood support system were mentioned as necessary to improve dietary diversity of infants.”* Please see page 15 for revision.

**REVIEWER #3**

**Comment 1**

In introduction part, dietary diversity and its relation with impaired growth need to be clearly mentioned. Dietary diversity and balanced meal are same?

**Response 1:** Please the introduction has been reviewed. Dietary diversity has been clearly explained in the second paragraph of introduction and did not mean balanced diet. Please see page 3

**Comment 2**

In methodology section

Why the study participants taken between 6-18 months and not 6 to 23 months which is standard.

Response 2: This paper was written from a larger study which sought to evaluate the effect of KOKOPLUS infant food supplement on children’s nutritional status as well as evaluating the effect of information about children’s current nutritional status on demand for KOKOPLUS (Please see study design under method section). From a random pilot study, majority of infants who were consuming this food supplement were aged between 6-18 months with a very low consumption rate for children above a year and half old (18 months). Therefore, to control for statistical disparities, the study decided to use the 6-18 months age group and this paper was written from the baseline data of this study.

**Comment 3**

24 hours recall method is not a proper estimation of dietary diversity specially here 9 food groups were taken as to maintain minimum dietary diversity which may not reflect the true diet habit for the participant.

**Response 3**

We understand the limitation of the use of 24-hour dietary recall. However, the 24-hour recall was used because according to Food and Agriculture Organization (FAO) Guidelines for measuring household and individual dietary diversity (see page 10), the reference period for the determining dietary diversity is the previous 24 hours. They further justified your point by saying that “using one 24-hour recall period does not provide an indication of an individual’s habitual diet, but it does provide an assessment of the diet at the population level and can be useful to monitor progress or target interventions”. Also, the 24-hour recall was used because according to the FAO, it is less subject to recall errors, less cumbersome for the respondent and also conforms to the recall time period used in many dietary diversity studies. Reference to this has been cited in the study (Page 5).

**Comment 4**

In result section,

table 1: The dietary diversity presence calculated by scoring 18 food groups intake, also test of significance applied taking these food groups as independent variables. Multicollinearity will be present for statistical test, thus cannot be applied.

**Response 4:** food groups were used to generate the dietary diversity; however, the food groups were not factored in the regression analysis and cannot affect multicollinearity. Also in the regression analysis, the Hosmer-Lemeshow goodness of fit was used to determine the fitness.

**Comment 5**

None of the table mentioned the total number of sample size (n) in title.

**Response 5:** Thanks for this comment. The total number of sample size has been added to Table 2 on page 9. The total sample size for the study was 1503 mother-infant pairs.

**Comment 6**.

The knowledge of mother regarding diet were shown in open ended but options given as dichotomous. The statements need to be modified.

**Response 6:** Please the participants were asked the open-ended questions however, all the answers they gave to each question was entered and sub-categorised as either correct or wrong and that was used to determine their nutrition knowledge. A broader explanation was given in page 5 of the methods

**Comment 7:**

The title and description didn't match in table 5. In title it is mentioned as dietary diversity. also, in methodology as presence of dietary diversity. But in binary regression the impression was made as presence of lower dietary diversity which should be mentioned in data analysis section and also the title needed to be changed.

**Response 7**: Generally, the aim and the method of the research was geared toward assessing factors associated with food consumption and dietary diversity among the infants, however it was noted that majority of them had very low dietary diversity. Hence, the binary regression was purposely conducted to know the factors responsible for the poor dietary diversity among the children though the title was not focused on poor dietary diversity.

**Comment 8:**

In supplementary table 2, how the test of significance was applied is not clear as the table had 3 variables.

**Response 8**: Please in supplementary table 1 and 2, because three variables (age group, food group and dietary diversity) were analysed, the Fisher’s Exact test was reported rather than the Chi square value. This has been stated clearly in the description of both supplementary table 1 and 2.
