# Supplementary material for: Factors associated with food consumption and dietary diversity among infants aged 6–18 months in Ashanti Region, Ghana
Source: PLoS One. 2023 Nov 30;18(11):e0294864. doi: 10.1371/journal.pone.0294864 (PMC10688859; doi:10.1371/journal.pone.0294864)
Supplement: Attachment — Submitted filename: Comments to authors_2.docx [file pone.0294864.s004.docx]

1. In the Materials and Methods section of the manuscript, keep only "Dependent variable:" and "Independent variable:" labeled as point. and remove the word "Data collection." Remove "socio-demographic," "complementary feeding knowledge," "complementary feeding practices," "child morbidity," and "dietary diversity" after "Dependent variable:" and "Independent variable:
2. In the "Strengths and Limitations of the study" section, remove the phrases "a very large sample size and" and "with a relatively smaller sample size" from the first sentence.
3. Check and very ole literatures should be replaced by the updated ones.
4. Include DOI or link to each reference.
