# Supplementary material for: Factors associated with food consumption and dietary diversity among infants aged 6–18 months in Ashanti Region, Ghana
Source: PLoS One. 2023 Nov 30;18(11):e0294864. doi: 10.1371/journal.pone.0294864 (PMC10688859; doi:10.1371/journal.pone.0294864)
Supplement: Attachment — Submitted filename: Response to Reviewer 2.docx [file pone.0294864.s005.docx]

Department of Biochemistry,

College of Science, KNUST,

Kumasi, Ghana.

Pradip Chouhan, PhD

Academic Editor

PLOS ONE

Dear Academic editor,

**POINT-BY-POINT REVISIONS IN RESPONSE TO REVIEWERS’ COMMENTS**

In line with the reviewer’s comments, the following revisions have been made to the manuscript ID: PONE-D-23-17357 entitled ‘Factors associated with food consumption and dietary diversity among infants aged 6-18 months in Ashanti Region Ghana’ by Godwin Opoku Agyemang, Samuel Selorm Attu, Reginald Annan, Satoru Okonogi, Takeshi Sakura and Odeafo Asamoah-Boakye.

Below is our submission;

**Response to Reviewer 2**

1. In the Materials and Methods section of the manuscript, keep only "Dependent variable:" and "Independent variable:" labeled as point. and remove the word "Data collection." Remove "socio-demographic," "complementary feeding knowledge," "complementary feeding practices," "child morbidity," and "dietary diversity" after "Dependent variable:" and "Independent variable:

**Response:** Please the Materials and Methods section of the manuscript have been revised accordingly. The dependent and independent variable have clearly been stated. The above said words have been removed accordingly. Please kindly refer to page 4 for corrections made.

2. In the "Strengths and Limitations of the study" section, remove the phrases "a very large sample size and" and "with a relatively smaller sample size" from the first sentence.

**Response**: Please the above phrases have been removed from the “Strengths and limitations” section on page 13.

3. Check and very ole literatures should be replaced by the updated ones.

**Response:** References in the study have been revised. Very old references are replaced with new ones with majority spanning from the past 6 years and few in the past 10 years.

4. Include DOI or link to each reference.

**Response**: Please a DOI has been added to majority of the references except those that had no DOI or link to them.
