# Supplementary material for: Factors associated with food consumption and dietary diversity among infants aged 6–18 months in Ashanti Region, Ghana
Source: PLoS One. 2023 Nov 30;18(11):e0294864. doi: 10.1371/journal.pone.0294864 (PMC10688859; doi:10.1371/journal.pone.0294864)
Supplement: Attachment — Submitted filename: Response to Reviewers.docx [file pone.0294864.s007.docx]

Department of Biochemistry,

College of Science, KNUST,

Kumasi, Ghana.

Sandra Boatemaa Kushitor, Ph.D.

Academic Editor

PLOS ONE

Dear Academic editor,

**POINT-BY-POINT REVISIONS IN RESPONSE TO REVIEWERS’ COMMENTS**

In line with the academic editor’s comments, the following revisions have been made to the manuscript ID: PONE-D-23-17357-R2 entitled ‘Factors associated with food consumption and dietary diversity among infants aged 6-18 months in Ashanti Region Ghana’ by Godwin Opoku Agyemang, Samuel Selorm Attu, Reginald Annan, Satoru Okonogi, Takeshi Sakura and Odeafo Asamoah-Boakye.

Below is our submission;

**Response to Academic Editor**

**Comment**: Include the implication or/and recommendation (Abstract section)

Response: Please the abstract has been reviewed to include the recommendations for the study (Page 2).

**Comment:** Please move this to the previous section (Materials and method section, page 4)

**Response:** This part has been removed and added to the previous section under the materials and methods section (page 4)

Comment: You dont have to report both the yes and the nos. the readers will appreciate this table better if you only report the percentage saying yes to the food item; and present all the food items as part of one column.

**Response:** Table 1 has been reconstructed based on the suggestion above. The table is currently reporting the infants who consumed the foods and its relationship on dietary diversity (Page 7).

Comment : Do not mix uppercase with lower cases. be consistent

**Response:** Please this point has been addressed in the manuscript (Page 7)

**Comment:** Do not let the words of one cell enter into another cell. What is the cronbach alpha value of this scale. please did you check whether the tool is culturally applicable to the Ghana's context.

Response: Please this point has been addressed (Page 11). The tables have been reconstructed to prevent overlapping of each other. This tool is applicable is culturally applicable in the Ghanaian certain as it has been applied in other published studies in Ghana.

**Comment:** Several independent variables have been left out. Please explain this in the data analysis section.

Response: Please this point has clearly been explained in the data analysis. The relationship between food consumption by age and dietary diversity has been attached as a supplementary table, though significance were seen.
